# Climate change impacts on *Xanthium strumarium* distribution: Integrating species distribution models with rhizosphere microbiome analysis in China

**DOI:** 10.1371/journal.pone.0351471

**Published:** 2026-08-21

**Authors:** Jinyang Dong, Liyan Zhang, Xiaoli Wang, Yanru Zhang, Mengzhe Zhu, Haiyan Jiang

**Affiliations:** Department of Forest Protection, College of Forestry, Inner Mongolia Agricultural University, Hohhot, Inner Mongolia, China; Shandong Normal University, CHINA

## Abstract

Global environmental changes increasingly alter species distributions, yet their effects on plants serving both ecological and economic functions remain inadequately explored. We examined *Xanthium strumarium*, a species with medicinal and invasive properties, throughout China using integrated approaches: species distribution modeling (Biomod2), niche analysis (Ecospat), and rhizosphere microbiome profiling (Tax4Fun). Our findings demonstrate that human footprint index (66.6% variable importance), elevation, and topographic slope primarily determine current distribution patterns. Future climate scenarios predict habitat expansion of 8.9–28.6%, identifying high-risk invasion zones primarily concentrated in Yunnan, Guangdong, and Inner Mongolia provinces. Although niche overlap analysis indicates high conservatism (Schoener’s D = 0.8986–0.9338), ecological adaptability shows a modest decline under elevated emission scenarios. Rhizosphere bacterial assemblages, characterized by Proteobacteria dominance and nitrogen-cycling taxa enrichment (*Nitrospira*, *Verrucomicrobia*), facilitate adaptation through enhanced metabolic pathways and environmental stress responses, promoting establishment in anthropogenically disturbed environments. Our results underscore the interactive effects of climate-mediated range shifts and microbiome-assisted resilience mechanisms underlying *X. strumarium’s* invasive potential. This research offers essential guidance for managing dual-function species, emphasizing integrated strategies that consider both anthropogenic pressures and microbial associations in conservation planning under accelerating global change.

## 1 Introduction

Climate change has emerged as one of the most significant environmental challenges facing humanity in the 21st century, affecting ecosystems, biodiversity, and species distribution across the globe [1–4]. This phenomenon is no longer a distant concern but a current reality with profound implications for both natural and human systems. Rising temperatures, altered precipitation patterns, and more frequent extreme weather events are increasingly reshaping ecosystems and altering the habitats of numerous species [5–7]. These changes are especially impactful on species distributions, as many plants and animals are forced to migrate, adapt, or face local extinction. The ability of species to respond to these shifts is not only dependent on the changes in temperature and precipitation, but also on the broader interactions within the ecosystem, such as plant-microbe relationships, and human-driven landscape transformations [8–10].

*Xanthium strumarium,* a plant species native to parts of Asia with concentrations in China and India that has spread globally to North and South America, Africa, and Europe, embodies a paradoxical nature—it is both valued in traditional medicine for its anti-inflammatory and analgesic properties and recognized as an aggressive invasive species threatening ecosystems in many regions [11–16]. In Traditional Chinese Medicine, its fruit, known as *Cang’erzi*, is used for its anti-inflammatory and analgesic properties to treat conditions like nasal congestion, headaches, and skin disorders [17]. This medicinal use gives the plant significant cultural and economic value. At the same time, it poses serious challenges to Chinese agriculture. The plant invades farmlands, reducing the yield of crops such as soybeans and cotton. Its spiny burrs contaminate sheep’s wool, which lowers its commercial value, and the plant can be toxic to livestock [18]. As a ruderal species, *X. strumarium* thrives in disturbed environments, outcompeting native vegetation and altering ecosystem dynamics [18–20]. It is well known for its rapid spread, especially in disturbed habitats such as agricultural fields and urban landscapes, where it competes for resources with native flora [21,22]. In China, the effects of climate change are particularly pronounced due to the country’s vast geographical expanse and diverse ecological zones, ranging from arid deserts in the northwest to subtropical forests in the south [23]. Home to over 30,000 plant species and a critical hub of global biodiversity, China faces unique challenges as shifting climates threaten its rich ecosystems and agricultural systems [24,25]. As a result, species distributions in China are undergoing significant transformations, with invasive species like *X. strumarium* exploiting these changes to expand their range, particularly in disturbed environments such as farmlands and urban fringes. Understanding these dynamics within the Chinese context is essential for predicting ecological shifts and informing conservation and management strategies.

Climate change has far-reaching consequences on species distributions, not only because of direct changes in climate parameters such as temperature and precipitation, but also because of the indirect effects resulting from the interactions between plants and their environments. For example, soil microbial communities, recognized as key drivers of plant growth, nutrient uptake, and stress tolerance, are highly sensitive to climatic changes [26,27]. Recent research has increasingly recognized the role of microbes in shaping plant species’ ecological success [28–32]. Climate-induced changes in temperature and precipitation can alter microbial community composition and functions, affecting how plants respond to abiotic stressors and how they interact with other species. In turn, these changes can modify plant competition dynamics, nutrient cycling, and ecosystem processes, all of which are critical to understanding the future distribution of species like *X. strumarium*.

The relationship between plants and their microbial symbionts is a pivotal, yet often overlooked, aspect of understanding plant distribution under climate change. Microbes in the soil and rhizosphere play essential roles in facilitating nutrient uptake, enhancing plant tolerance to stressors such as drought and soil salinity, and even protecting plants from pathogens [33–37]. These microbial communities are not static; they evolve and adapt to changing environmental conditions, and their composition can influence how plants cope with the challenges posed by a warming world. For example, some plant species may benefit from symbiotic relationships with beneficial microbes that help them thrive in conditions of water scarcity or higher temperatures [38,39]. Conversely, other species may struggle as their microbial partners are unable to adapt quickly enough to rapid environmental shifts [40,41]. Understanding these complex interactions is vital for predicting how plant species like *X. strumarium* will fare in future climate scenarios, especially considering its status as both a medicinal plant and an invasive species.

Moreover, climate change is intricately intertwined with human activities that are fundamentally altering the landscape and ecosystem dynamics [42,43]. Agricultural expansion, urbanization, deforestation, and the intensification of land-use practices all contribute to habitat destruction, fragmentation, and the creation of novel ecological niches [44–46]. These anthropogenic activities not only alter the physical landscape but also influence the ecological processes that govern species distributions. *X. strumarium* has shown a remarkable ability to colonize disturbed habitats, which are often the result of human activities [47]. The spread of *X. strumarium* has been closely linked to agricultural intensification, where the plant benefits from disturbed soil conditions and the increased availability of resources in disturbed landscapes [48]. Additionally, urbanization has facilitated its expansion by providing fragmented, disturbed environments that allow for its persistence and spread [49,50].

Human-driven habitat fragmentation and alteration can exacerbate the effects of climate change on species [51–53]. Fragmented habitats may reduce the overall fitness of species by isolating populations, thereby limiting gene flow and reducing genetic diversity [54,55]. This can make species more vulnerable to climate-induced stresses, such as changes in temperature, precipitation, or the availability of resources [56]. Furthermore, human activities have introduced new challenges for species like *X. strumarium*, which are already adapting to shifting climates. For instance, land-use changes can disrupt natural plant-microbe interactions, alter nutrient availability, and reduce the resilience of ecosystems [57]. In this sense, human activities act as both direct and indirect drivers of species distribution shifts, further compounding the challenges posed by climate change.

To better understand how *X. strumarium* will respond to climate change, one of the most powerful tools at our disposal is species distribution modeling (SDM). SDMs are computational tools used to predict the potential distribution of species based on environmental variables, such as temperature, precipitation, and soil characteristics [58–60]. These models can simulate current and future species distributions under different climate scenarios, providing valuable insights into how species may fare under future climate conditions. Among the various modeling approaches, Biomod2 has emerged as a robust and flexible framework for SDM [61–65]. Biomod2 integrates multiple modeling algorithms, including Generalized Linear Models (GLMs), Random Forests (RF), Artificial Neural Networks (ANNs), and Support Vector Machines (SVMs), to create an ensemble model that combines the strengths of each algorithm [66,67]. This ensemble approach reduces the uncertainty inherent in individual models, making Biomod2 particularly valuable for predicting species distributions under complex environmental scenarios [68,69].

To complement the predictive power of SDMs, tools like Ecospat are invaluable for analyzing niche dynamics and species-environment interactions [70]. Ecospat provides a framework for assessing niche overlap and shifts, offering insights into how *X. strumarium* might adapt to future climate scenarios [71]. By evaluating the ecological niche of *X. strumarium* under current and projected climates, Ecospat helps identify potential areas of habitat suitability and the degree of niche conservatism or shift.

The role of soil microorganisms in influencing plant distribution and adaptation is increasingly acknowledged [72–74]. The rhizosphere, enriched by root exudates and microbial interactions, is crucial for plant health and resilience [75]. To gain a deeper understanding of the microbial community’s role, Tax4Fun serves as an invaluable resource [76]. Tax4Fun is a bioinformatics tool that predicts the functional capabilities of microbial communities by translating 16S rRNA gene sequence-based taxonomic data into functional profiles, allowing researchers to infer the communities’ metabolic potential [77,78].

This study aims to investigate the potential impacts of climate change on the distribution of *X. strumarium* across China by utilizing Species Distribution Modeling (SDM) tools, including Biomod2. The research will evaluate how shifts in environmental variables—such as temperature and precipitation—influence the plant’s distribution patterns within Chinese ecoregions. To elucidate the species’ adaptive capacity under projected climate scenarios, niche dynamics and species-environment interactions will be analyzed using Ecospat. Concurrently, Tax4Fun will be employed to characterize the functional profiles of soil microbial communities and their role in shaping plant-microbe symbiosis critical for the plant’s ecological success in China’s diverse landscapes. By integrating these multidimensional analyses, this work seeks to deliver a holistic understanding of the plant’s future ecological and economic implications, particularly considering its dual role as a medicinal resource and an aggressive invasive species within the Chinese context.

## 2 Materials and methods

### 2.1 Data sources and processing

**Fig 1:**
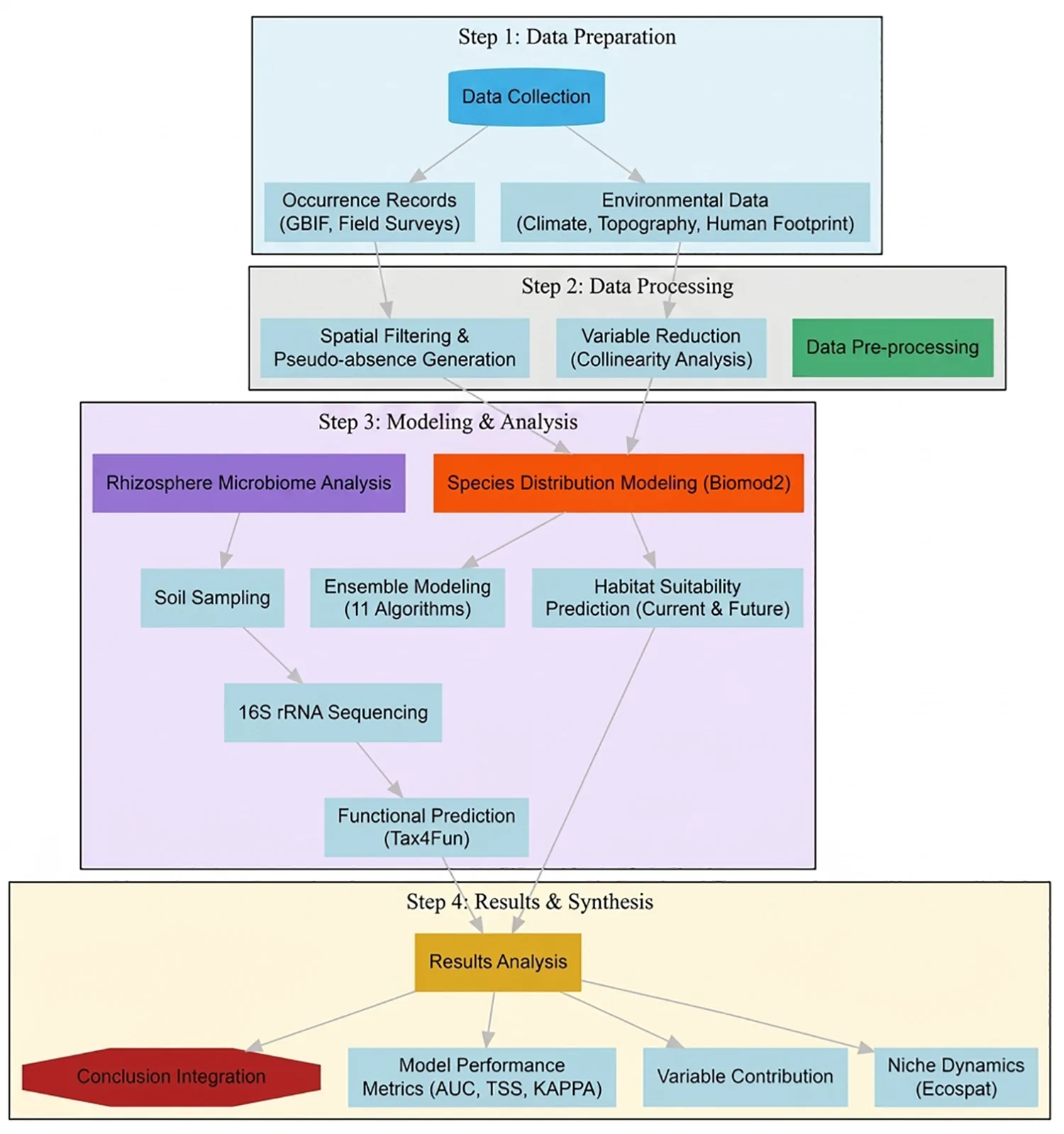
An integrated methodological framework for analyzing climate change impacts on *Xanthium strumarium* distribution and its rhizosphere microbiome.

This study employs a comprehensive, multi-step methodological framework to investigate the potential impacts of climate change on the distribution of *X. strumarium* and the role of its rhizosphere microbiome. As illustrated in the methodological flowchart (Fig 1), the research workflow is divided into four main stages: Data Preparation, Data Processing, Modeling & Analysis, and Results & Synthesis.

The Data Preparation stage focused on collecting two core datasets: species occurrence records from GBIF and field surveys, and a set of environmental variables that includes climate, topography, and human activity data. Subsequently, in the Data Processing stage, these datasets were refined through spatial filtering and pseudo-absence generation for occurrence data, and collinearity analysis to select the most impactful environmental variables.

The core of our research, Modeling & Analysis, was conducted in parallel. Species distribution modeling was performed using the Biomod2 ensemble framework, while rhizosphere microbial analysis involved soil sampling followed by 16S rRNA gene sequencing and functional prediction using Tax4Fun. Finally, the Results & Synthesis stage integrated all findings, including model performance metrics and niche dynamics analysis, to provide a holistic understanding of the species’ response to climate change. The following sections provide a detailed explanation of each step in this workflow.

**Fig 2:**
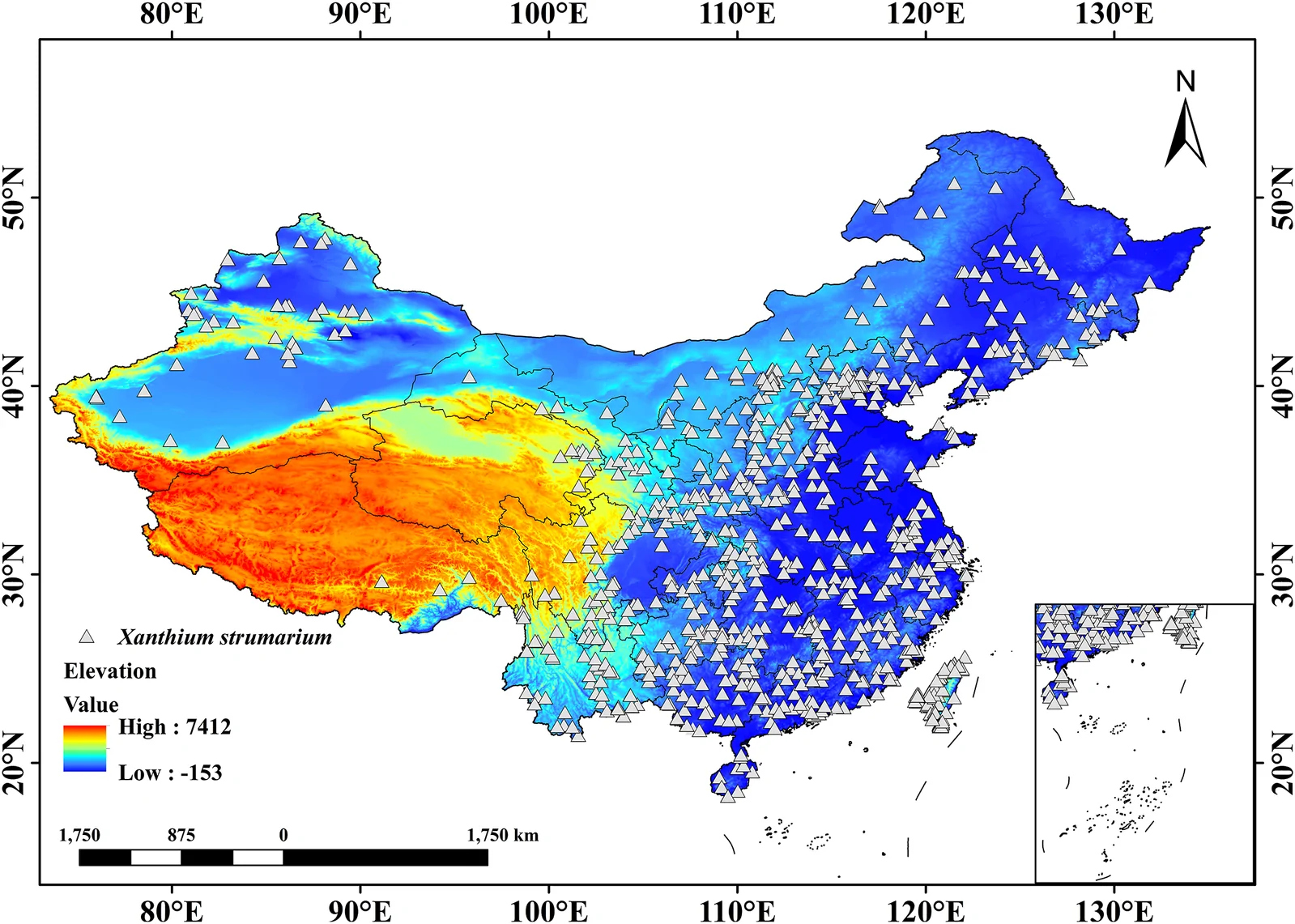
Geographic distribution of *Xanthium strumarium* occurrence records in China. Each grey triangle represents a spatially filtered occurrence point used for modeling in this study. The background color gradient illustrates the elevation across the country, with high-altitude regions.

Distribution data for *X. strumarium* were compiled from the Global Biodiversity Information Facility (GBIF) and complemented by field surveys conducted in Inner Mongolia Autonomous Region, China, between 2022 and 2024 [79]. To mitigate spatial sampling bias and enhance model reliability, we implemented a 5 km^2^ spatial filtering protocol on the initial 2,288 occurrence records using the “Spatially Rarefy Occurrence Data” tool within ArcGIS 10.8 (Environmental Systems Research Institute, Redlands, CA, USA). This preprocessing step effectively reduced spatial autocorrelation while preserving ecological representativeness, yielding 1,689 spatially independent occurrence points suitable for subsequent distribution modeling analyses (Fig 2).

For the present research, 23 environmental variables were selected to comprehensively model the habitat suitability of *X. strumarium*. This selection, comprising 19 climatic variables, three topographic variables, and one human activity variable, was guided by their direct ecological relevance to this ruderal species, which is known to colonize disturbed habitats. The three topographic variables of elevation, slope, and aspect were included to define the species’ physical landscape constraints, with elevation acting as a primary limiting factor for altitudinal survival, slope identifying the low-gradient terrains on which the species thrives, and aspect influencing the microclimatic conditions that further refine habitat suitability [14]. The human activity variable was incorporated to account for anthropogenic activities that create disturbed habitats, while the Normalized Difference Vegetation Index (NDVI) was chosen as a key biotic proxy to identify these vulnerable sites based on vegetation cover and potential competition [80]. Climatic and elevation data were obtained from WorldClim at a 5-km resolution [81]. Topographic variables, including slope and aspect, were derived from elevation data using the Surface Analysis tool in ArcGIS 10.8. Human activity data were sourced from the third version of the Global Human Modification dataset at a 1-km resolution [82]. This study employs the Moderate Resolution Imaging Spectroradiometer (MODIS) monthly NDVI (MOD13A3) to characterize vegetation growth dynamics in the study area [83]. The dataset integrates multi-temporal remote sensing data processed through Maximum Value Compositing (MVC) algorithm, generating spatio-temporally continuous vegetation indices. With a spatial resolution of 1 km × 1 km and annual temporal coverage, this product effectively mitigates cloud contamination effects while highlighting vegetation phenological characteristics during peak growing seasons. The administrative boundary data used for creating the study area base maps was obtained from Natural Earth (https://www.naturalearthdata.com/).

Future climate projections were sourced from the BCC-CSM2-MR model, developed by the Beijing Climate Center and widely recognized for its strong performance in studies exploring vegetation distribution across China [84,85]. This model provided data for three climate scenarios, SSP1–2.6, SSP2–4.5, and SSP5–8.5, which correspond to progressively higher carbon emission levels. For each scenario, data were retrieved for two time periods, spanning 2041–2060 and 2061–2080, at a spatial resolution of 5 km.

The twenty-three environmental variables obtained above were imported into ArcGIS 10.8. The “Extract by Mask” tool was used to crop the data to the geographical extent of China, and bilinear interpolation was applied to resample the resolution of the environmental variables to a standardized 5 km. The 5 km scale was deliberately chosen for its methodological consistency in integrating diverse predictor variables, its ecological appropriateness for capturing the broad-scale distribution patterns of a widespread species like *X. strumarium*, and its capacity to minimize the influence of fine-scale environmental noise and spatial inaccuracies in the occurrence data [86,87]. This process ensures the necessary data consistency for subsequent spatial analysis and model construction.

**Fig 3:**
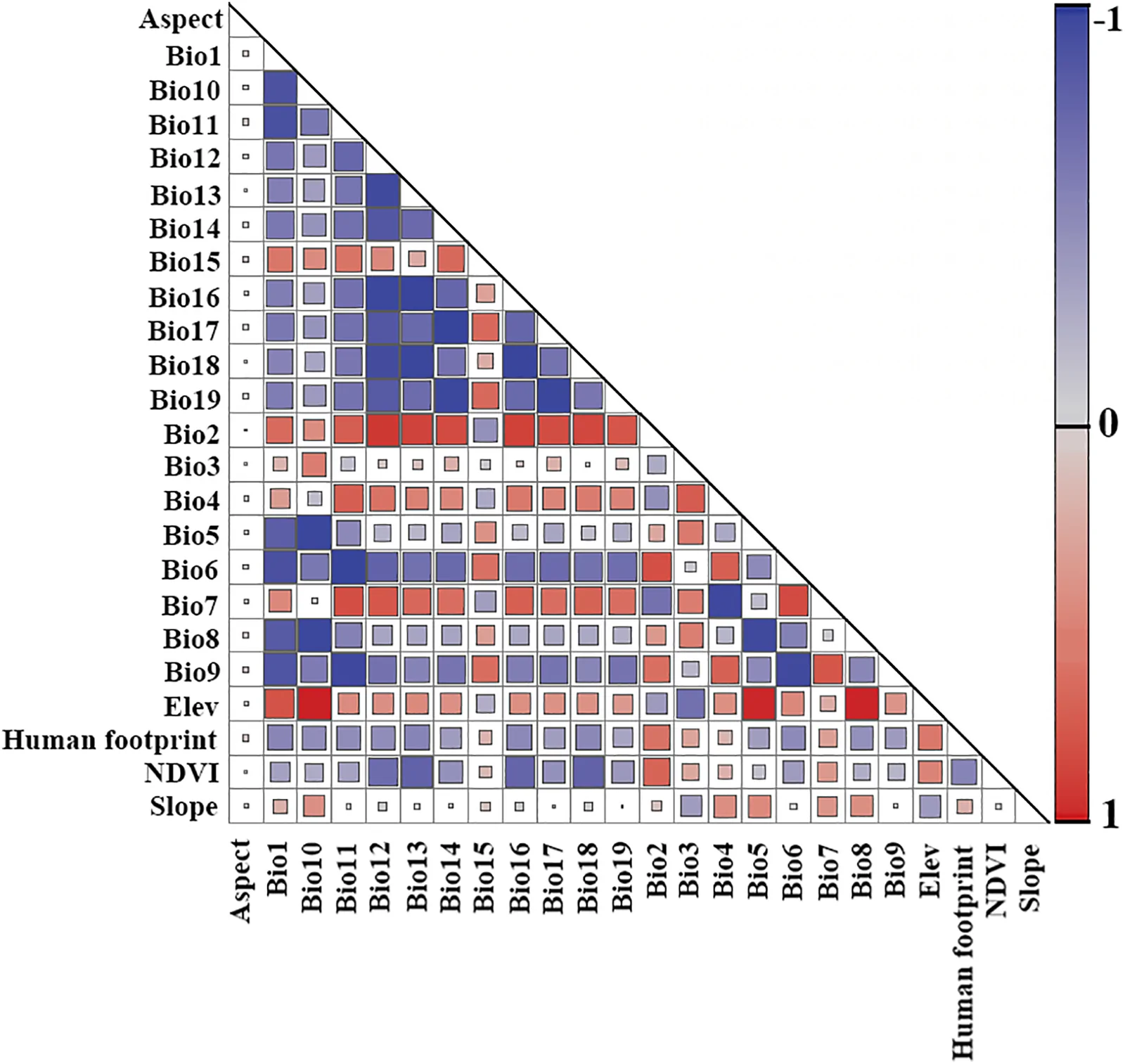
Pearson correlation matrix for the initial 23 environmental variables. The correlogram displays the Pearson correlation coefficients between pairs of variables. The color and size of the squares indicate the strength and direction of the correlation: blue squares represent a positive correlation, red squares represent a negative correlation, and the intensity of the color corresponds to the magnitude of the coefficient (from −1 to +1). This analysis was conducted to identify and remove highly collinear variables for subsequent modeling.

Multiple factors may exhibit collinearity, potentially influencing the outcomes of the model [88]. To mitigate the problem of collinearity among variables, a stepwise variable reduction approach was employed. All environmental variables were imported into R, and the Pearson correlation coefficients between them were calculated using the “terra” package. These variables were then loaded into “biomod2” to filter out those with a contribution greater than zero. For pairs of filtered variables with a correlation coefficient exceeding 0.8, the variable with the higher contribution was retained [89]. This process culminated in the selection of 13 variables (Fig 3 and Table 1).

**Table 1:** Environmental variables for the models.

Category	Variable	Description	Unit
Climate	Bio2	Mean diurnal range	°C
	Bio3	Isothermality	\
	Bio4	Temperature seasonality	\
	Bio8	Mean temperature of wettest quarter	°C
	Bio10	Mean temperature of warmest quarter	°C
	Bio11	Mean temperature of coldest quarter	°C
	Bio15	Precipitation seasonality	\
	Bio17	Precipitation of driest quarter	mm
Topography	Elev	Elevation	m
	Aspect	Aspect	°
	Slope	Slope	°
Human	Human footprint	Human footprint	\
Vegetation	NDVI	Normalized difference vegetation index	\

### 2.2 Models construction and changes in ecological niches

To build a reliable prediction of the species’ potential habitat, this study used an ensemble approach that combines the outputs of 11 different modeling algorithms. These specific algorithms were chosen because they represent a wide variety of different methods for predicting species distribution. The selection includes traditional statistical models, various machine learning techniques, and other common ecological modeling approaches. By using such a diverse set of tools, we can capture different aspects of the relationship between the species and its environment. This strategy ensures that the final prediction is comprehensive and not overly dependent on the specific assumptions of any single method, which helps to reduce uncertainty and increase the overall accuracy of the results.

The 11 algorithms used in the modeling framework, each run 10 times for robustness [65], were the Artificial Neural Network (ANN), Classification Tree Analysis (CTA), Flexible Discriminant Analysis (FDA), Generalised Additive Model (GAM), Generalised Boosting Model (GBM), Generalised Linear Model (GLM), Multivariate Adaptive Regression Spline (MARS), Maximum Entropy (MAXENT), Random Forest (RF), Surface Range Envelope (SRE), and eXtreme Gradient Boosting (XGBOOST).

Community occurrence data were combined with an equal number of randomly generated pseudo-absences, split into 75% training and 25% test sets via random sampling. Pseudo-absences were generated using the ‘BIOMOD_FormatingData’ function, a method proven effective for capturing potential distributions compared to real absence data [90]. Individual model outputs were integrated into an ensemble model (EM) via a weighted average algorithm, enhancing predictive precision [91]. Habitat suitability was classified into four categories corresponding to potential invasion risk levels: No Risk Zone (0–0.25), Low-Risk Zone (0.25–0.50), Medium Risk Zone (0.50–0.75), and High-Risk Zone (0.75–1.0), based on model probability outputs.

Model accuracy was assessed using three metrics: the Area Under the Receiver Operating Characteristic curve (AUC), True Skill Statistic (TSS), and Kappa coefficient, with thresholds of ROC > 0.9, TSS > 0.85, and Kappa > 0.85 indicating high performance [66,92,93].

Under current climate conditions, the distribution points of *X. strumarium* were identified, and a 1-degree buffer zone surrounding these points was established as the background for analysis. For predicted future climate scenarios, background points were determined based on suitable habitat areas projected by an ensemble model. By integrating these distribution points with diverse climate datasets, the “ecospat” R package was employed to evaluate and quantify the niche overlap of *X. strumarium* under both current and future climate conditions [70]. This package facilitated the visualization of niche dynamics and the computation of the Schoener’s D niche overlap parameter (observed value), which ranges from 0 (no overlap) to 1 (complete overlap) [94]. This methodology enables a comprehensive analysis and assessment of the potential effects of climate change on the ecological niche of *X. strumarium*.

### 2.3 16S rRNA gene sequencing of rhizosphere microbial communities

Soil samples were collected from the rhizosphere of *X. strumarium* across five distinct habitat types in Inner Mongolia, China (Table 2). The selection of the five sampling sites in Inner Mongolia was strategically designed based on our preliminary Species Distribution Model (SDM) predictions. The SDM identified Inner Mongolia as a highly vulnerable emerging hotspot for X. strumarium expansion under future high-emission scenarios (e.g., SSP5–8.5). Furthermore, since the SDM revealed that the ‘Human Footprint Index’ is the dominant macro-environmental driver (contributing 66.6%), these five specific sites (representing urban green spaces, farmland edges, grasslands, montane forests, and temperate steppes) were chosen to reflect a gradient of anthropogenic disturbance. This strategic selection allows us to explore the micro-ecological mechanisms underlying the macro-scale distribution patterns. The soil samples were collected from public-access areas in Inner Mongolia, China, and did not involve any protected species or private lands.

**Table 2:** Rhizosphere soil sampling sites of *Xanthium strumarium.*

Site	Location	Coordinates	Habitat Type
ZD1	Yematu Village, Xincheng District, Hohhot	111°51′48.194″E, 40°55′52.378″N	Farmland Edge
ZD2	Hadamen National Forest Park	111°35′18.977″E, 41°01′04.115″N	Montane Forest
ZD4	Saihanwula National Nature Reserve	118°39′40.185″E, 44°15′41.225″N	Temperate Steppe
ZD5	East Campus, Inner Mongolia Agricultural University	111°43′00.034″E, 40°49′03.373″N	Urban Green Space
ZD6	Huanghuagou Grassland Cultural Resort	112°32′06.128″E, 41°08′28.420″N	Grassland

Sampling was conducted in July 2024, targeting five replicate *X. strumarium* plants per site. Rhizosphere soil (0–5 cm depth, ~ 500 g) was collected within 2 mm of roots using a sterile corer, with bulk soil con-trols sampled 1 m away. Samples were stored at 4°C, transported to the laboratory within 24 h, and subsampled for DNA extraction (stored at −80°C) and physicochemical analysis (air-dried, sieved <2 mm). Genomic DNA was extracted from 0.5 g soil using the DNeasy PowerSoil Kit (Qiagen, Germany), with quality verified by NanoDrop 2000 and gel electrophoresis [95–97].

The 16S rRNA V3–V4 region was amplified (primers 338F/806R), sequenced on an Illumina MiSeq (PE300), and processed in QIIME2 (v2022.8) with DADA2 for ASV identification. Taxonomy was assigned using SILVA (v138), and datasets were rarefied to 10,000 reads/sample. Microbial diversity (Shannon, Chao1, Bray-Curtis NMDS) was analyzed, and functional profiles were predicted with Tax4Fun (v1.0) against KEGG pathways, validated by shotgun metagenomics (Spearman’s ρ > 0.85). To test for significant differences in microbial community composition among different sample groups, we performed a PERMANOVA (Adonis) analysis based on the Bray-Curtis distance using the vegan package in R.

Tax4Fun is an R package designed for functional prediction of environmental samples, such as gut microbiota and soil, based on the 16S Silva database [78]. It offers high prediction accuracy and outperforms the PICRUSt functional prediction, particularly for complex environmental samples like soil. The functional prediction in Tax4Fun is achieved through a nearest-neighbor approach based on minimal 16S rRNA sequence similarity. Specifically, it extracts prokaryotic whole-genome 16S rRNA gene sequences from the KEGG database and uses the BLASTN algorithm to align them to the SILVA SSU Ref NR database (with a BLAST bit score >1500), constructing a relevant matrix. The KEGG database’s prokaryotic functional annotations, identified by UProC and PAUDA methods, are then mapped to the SILVA database for functional annotation. Sequenced samples are clustered into OTUs using SILVA database sequences as reference, allowing for functional annotation retrieval.

## 3 Results

### 3.1 Model precision assessment

**Fig 4:**
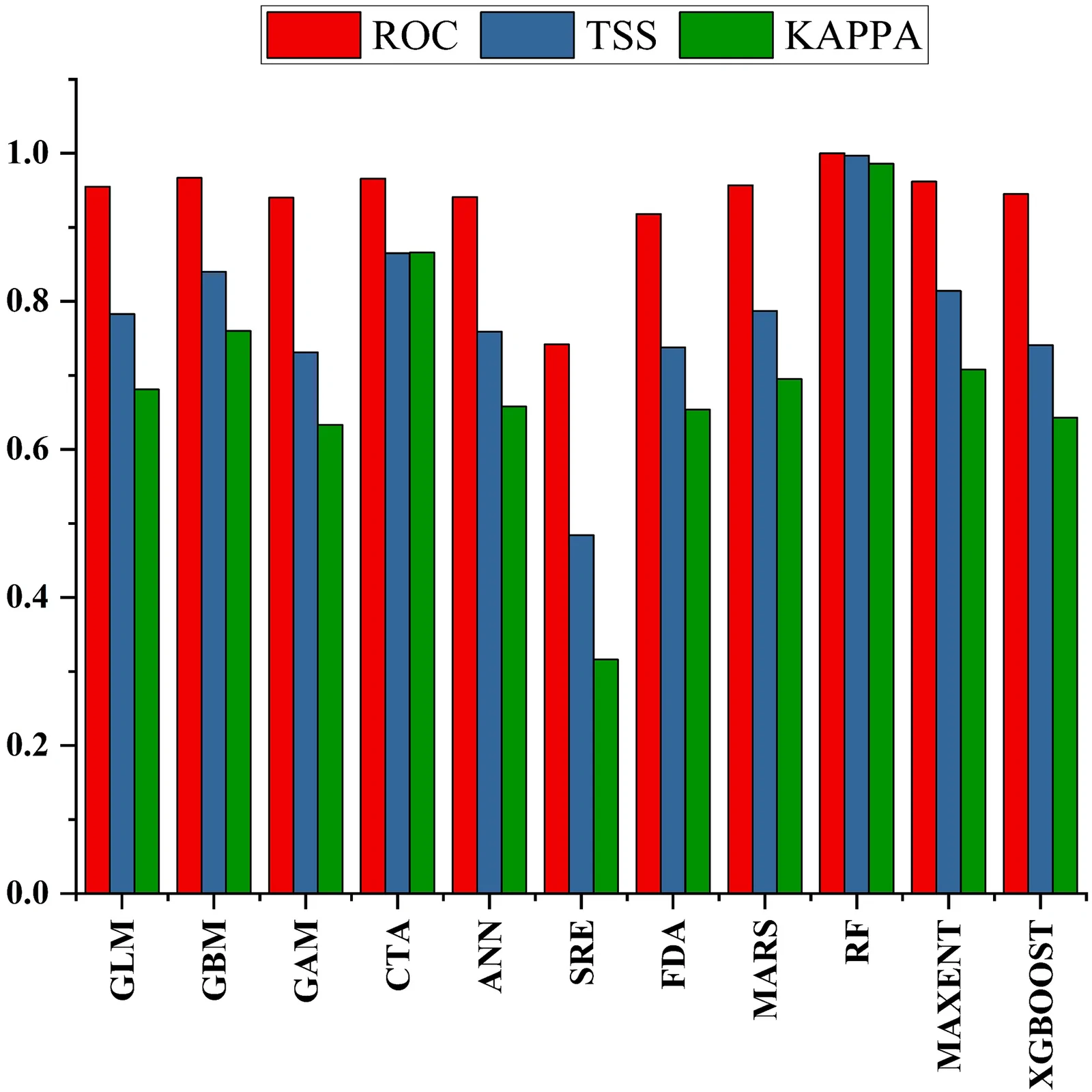
Performance evaluation of individual species distribution models. The bar chart compares the predictive performance of 11 different modeling algorithms. Performance is measured by three metrics: Area Under the Receiver Operating Characteristic curve (ROC; red bars), True Skill Statistic (TSS; blue bars), and Kappa coefficient (KAPPA; green bars). For all metrics, values closer to 1.0 indicate higher model accuracy. Model acronyms are listed along the x-axis, ANN: artificial neural network, CTA: classification tree analysis, FDA: flexible discriminant analysis, GAM: generalised additive model, GBM: generalised boosting model, GLM: generalised linear model, MARS: multivariate adaptive regression spline, MAXENT: maximum entropy, RF: random forest, XGBOOST: eXtreme gradient boosting, SRE: surface range envelope.

In this study, we conducted a comprehensive evaluation of the various predictive models used, focusing on their performance in terms of AUC, TSS, and Kappa values (Fig 4).

Regarding the AUC value, which measures the classification ability of a model, a value closer to 1 indicates superior predictive performance. Our analysis showed that the RF model achieved the highest AUC value of 1.0. Other models, such as GBM (0.967), MAXENT (0.962), and MARS (0.957), also yielded high AUC values near 0.96. The ANN and GLM models returned AUC values close to 0.95, while the SRE model exhibited a significantly lower AUC value of 0.742.

Next, we assessed the True Skill Statistic (TSS), which evaluates a model's discrimination ability independent of prevalence. TSS values range from −1 to +1, with values above 0.75 often considered to indicate excellent performance. The RF model achieved the highest TSS score (0.997). Several other models also returned TSS values above this threshold, including CTA (0.865), GBM (0.840), and MAXENT (0.814). The SRE model returned the lowest TSS value of 0.484.

The Kappa value assesses the consistency of a model’s predictions beyond random chance, with a value closer to 1 indicating stronger consistency. Among all models, RF demonstrated the highest Kappa value (0.986). Both CTA (0.866) and GBM (0.760) also returned high Kappa values. In contrast, the Kappa values for ANN and GLM were 0.658 and 0.681, respectively. The SRE model had a Kappa value of 0.316, the lowest among all models evaluated.

In conclusion, RF emerged as the most outstanding individual model. While all models demonstrated AUC values greater than 0.7, the SRE model was excluded from further consideration due to its consistently low performance across all metrics.

For the final ensemble model, we employed the mean of probabilities (EMmean) method, which calculates the average habitat suitability score from all selected individual models. This approach was chosen for its robustness and transparency. By averaging the outputs, the EMmean method effectively balances the predictions from the diverse set of algorithms and minimizes the biases inherent in any single model. This often leads to a more generalized and reliable forecast than more complex weighting schemes. The effectiveness of this method was confirmed by its high predictive accuracy, yielding an ensemble TSS value of 0.911, an AUC value of 0.971, and a Kappa value of 0.888.

### 3.2 Bioclimatic variable contribution

**Fig 5:**
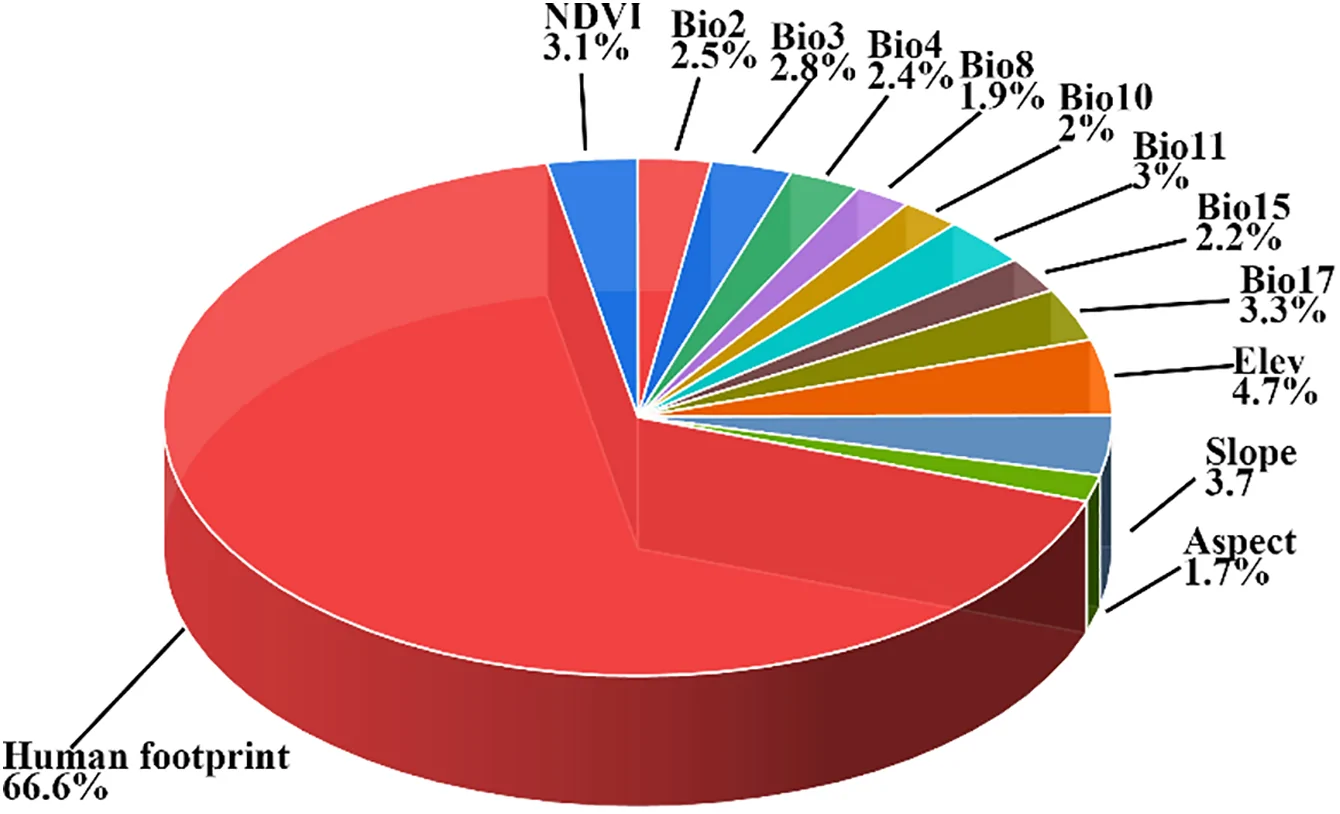
Relative contribution of environmental variables to the *Xanthium strumarium* distribution model. The pie chart illustrates the percentage of contribution from each of the 13 selected environmental variables in predicting the habitat suitability for *Xanthium strumarium*. The human footprint index was the most significant predictor, accounting for 66.6% of the model’s explanatory power.

The modeling results demonstrate the relative contributions of different environmental variables to *X. strumarium* (Fig 5), with human activity having the most significant impact, accounting for 66.6% of the contribution. Elevation and slope contribute 4.7% and 3.7%, respectively, indicating that topographic variables have a certain degree of influence on ecological processes. Additionally, vegetation cover (NDVI) and a series of bioclimatic variables have relatively smaller contributions but still provide necessary insights into environmental complexity.

### 3.3 Present potential geographic spread

**Fig 6:**
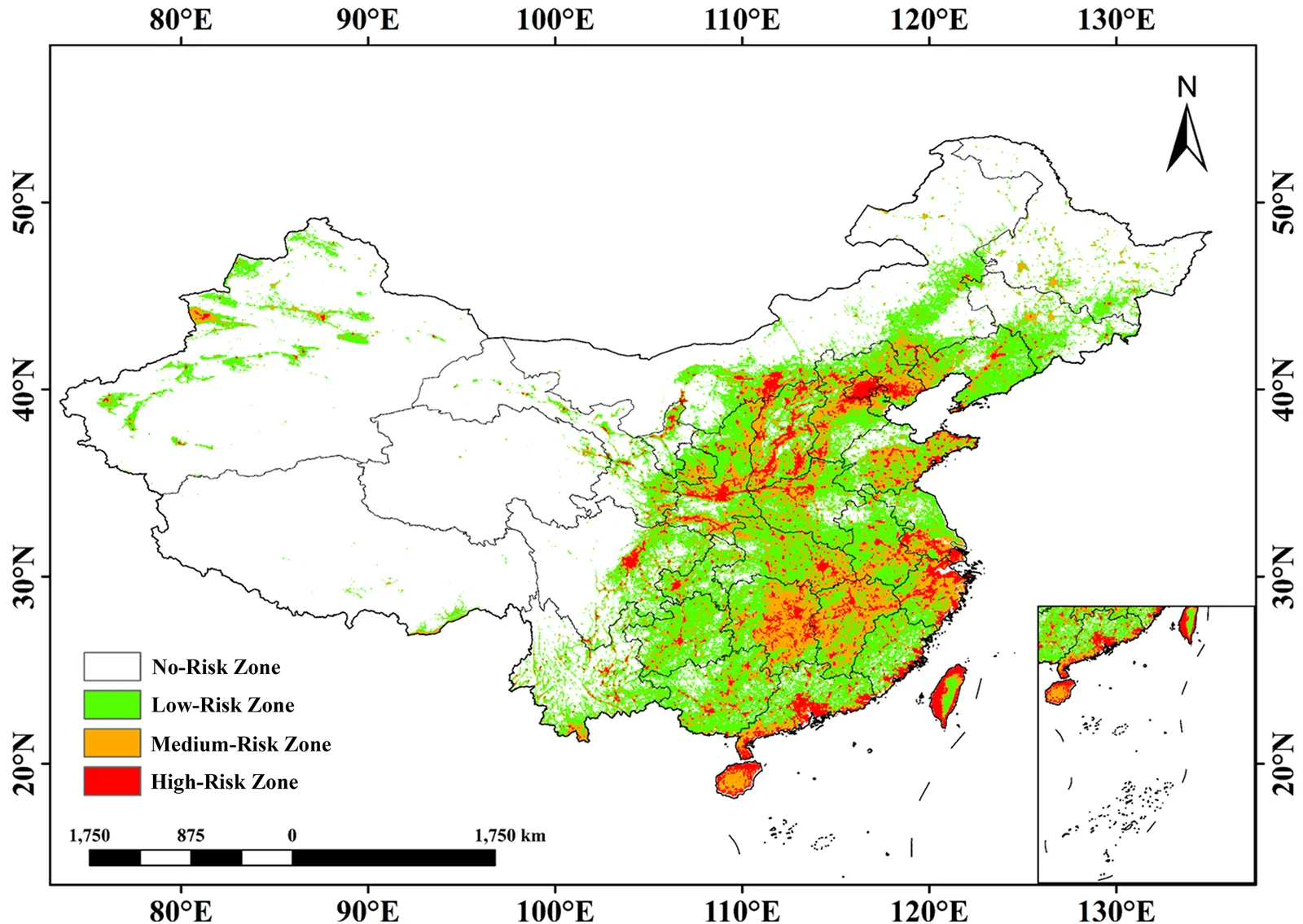
Predicted potential distribution of suitable habitats for *Xanthium strumarium* in China under current environmental conditions. This map, generated by the ensemble model, shows habitat suitability classified into four levels: No-Risk Zone (white), Low-Risk Zone (green), Medium-Risk Zone (orange), and High-Risk Zone (red).

The integrated modeling results indicate that the current primary potential suitable habitats for *X. strumarium* are predominantly distributed across southern, central, eastern, northeastern, and southeastern China, encompassing a total suitable area of 329.816 × 10^4^ km^2^ (Fig 6). Specifically, high-risk zones (36.871528 × 10^4^ km^2^) are principally concentrated in the following administrative regions: Inner Mongolia, Hebei, Tianjin, Beijing, Shandong, Henan, Shanxi, Hainan, Hunan, Hubei, Guangdong, and Taiwan, while scattered occurrences are observed in Xinjiang, Xizang, and Yunnan provinces.

**Fig 7:**
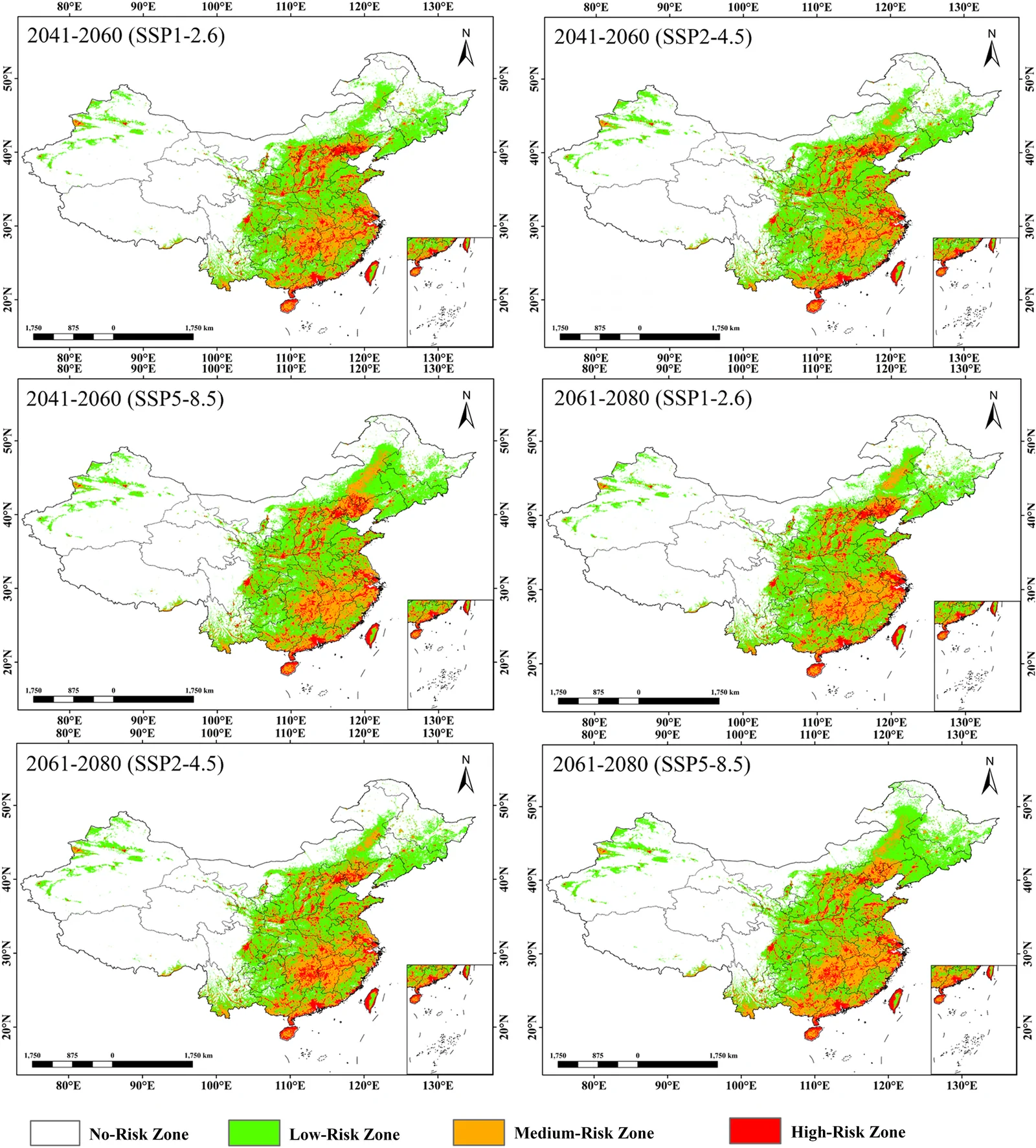
Projected future distributions of suitable habitats for *Xanthium strumarium* in China. The six maps show predicted habitat suitability for two future periods (2041–2060 and 2061–2080) under three Shared Socioeconomic Pathways (SSPs): SSP1–2.6 (low emissions), SSP2–4.5 (moderate emissions), and SSP5–8.5 (high emissions). The legend indicates the four suitability levels from No-Risk Zone (white) to High-Risk Zone (red).

Fig 6 shows the current suitable habitats for *X. strumarium* in China, and Fig 7 projects how these areas will expand under future climate scenarios.

This Table 3 and Fig 7 show the changes in the suitable growth areas of *X. strumarium* under different climate scenarios over the next few decades. In the coming decades, regardless of whether it is a low, medium, or high emission scenario (SSP1–2.6, SSP2–4.5, SSP5–8.5), the areas of low and moderately risk zones for *X. strumarium* are showing an increasing trend, especially under high emission scenarios, where the growth is more significant.

**Table 3:** Changes in the distribution area of *Xanthium strumarium* during different periods under different scenarios (10^4^ km^2^).

Period	Climate Scenario	Low-Risk Zone	Medium-Risk Zone	High-Risk Zone
Current		198.70486	94.239585	36.871528
2041-2060	SSP1–2.6	214.12327	121.80209	47.453126
	SSP2–4.5	214.8698	122.04167	46.569445
	SSP5–8.5	225.38889	134.07813	46.918404
2061-2080	SSP1–2.6	210.57292	127.92188	46.440973
	SSP2–4.5	209.90973	127.00695	46.953126
	SSP5–8.5	228.59375	141.42361	46.119792

### 3.4 Niche dynamics

**Fig 8:**
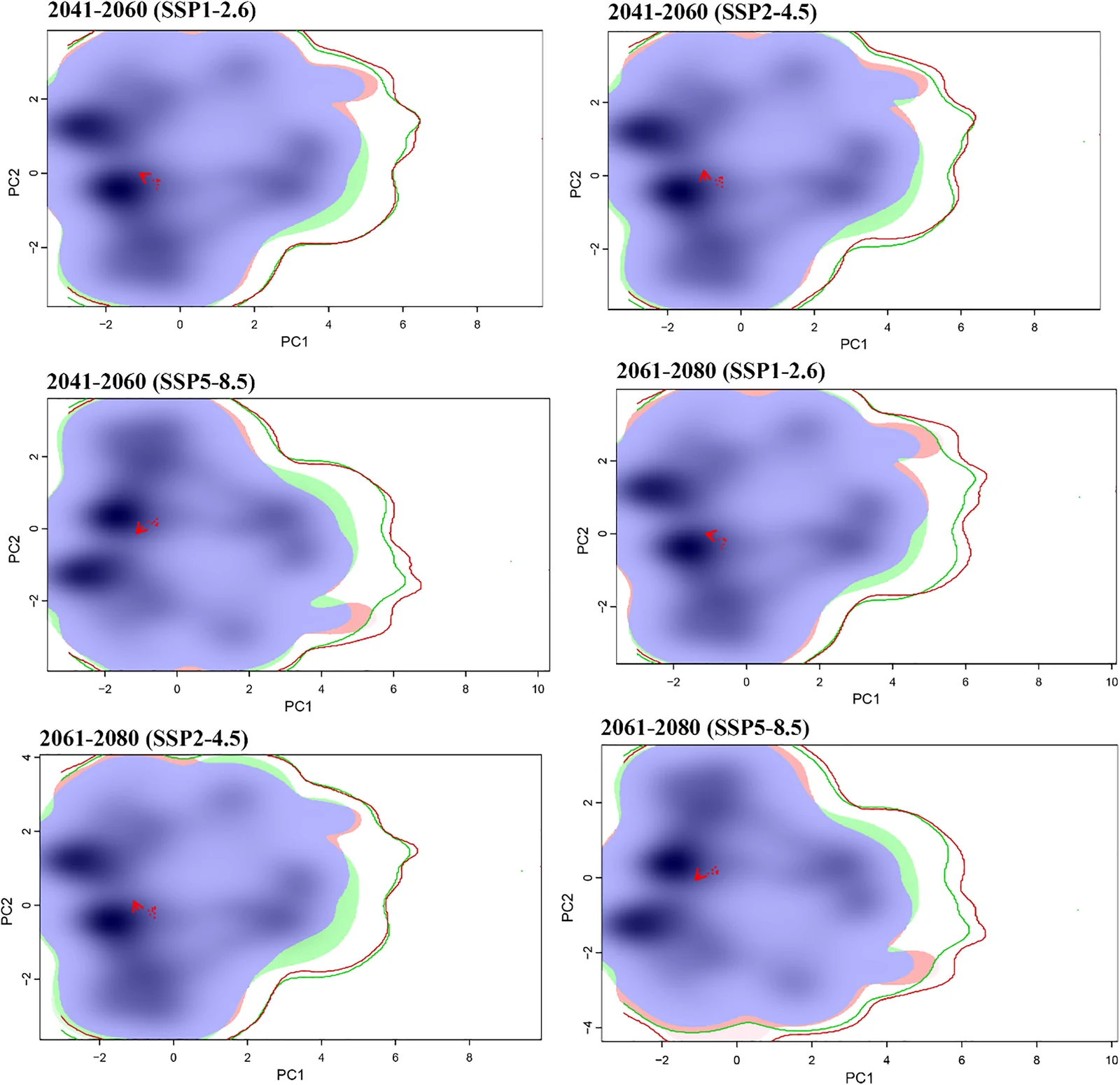
Niche dynamics of *Xanthium strumarium.*

The niche comparison analysis for *X. strumarium* across current and projected future climate scenarios reveals a substantial degree of ecological niche overlap, as quantified by Schoener’s D metric (Fig 8). Specifically, the overlap ranges from a minimum of 89.86% (Schoener’s D = 0.8986) between the current climate and the 2061–2080 period under the SSP5–8.5 scenario, characterized by high levels of greenhouse gas emissions, to a maximum of 93.38% (Schoener’s D = 0.9338) for the same timeframe under the more sustainable SSP1–2.6 pathway. Intermediate values include 93.20% (Schoener’s D = 0.9320) for the 2041–2060 period under SSP1–2.6, 93.17% (Schoener’s D = 0.9317) under SSP2–4.5, and 91.20% (Schoener’s D = 0.9120) under SSP5–8.5, while the overlap for 2061–2080 under SSP2–4.5 stands at 90.38% (Schoener’s D = 0.9038).

These plots visualize the ecological niche in a two-dimensional environmental space defined by the first two principal components (PC1 and PC2). The colors represent: niche stability (blue shading, overlap between current and future niches), niche unfilling (light green, part of the current niche not occupied in the future), and niche expansion (light red, new environmental space projected to be occupied). The red and green solid lines delineate 100% of the available environmental space for the current and future periods, respectively. The extended contours in the bottom-left corner of the SSP5–8.5 plots represent the expansion and shift of the available background climate space under the extreme emission scenario, rather than a statistical artifact.

### 3.5 Rhizosphere microbial abundance in *Xanthium strumarium* habitats

**Fig 9:**
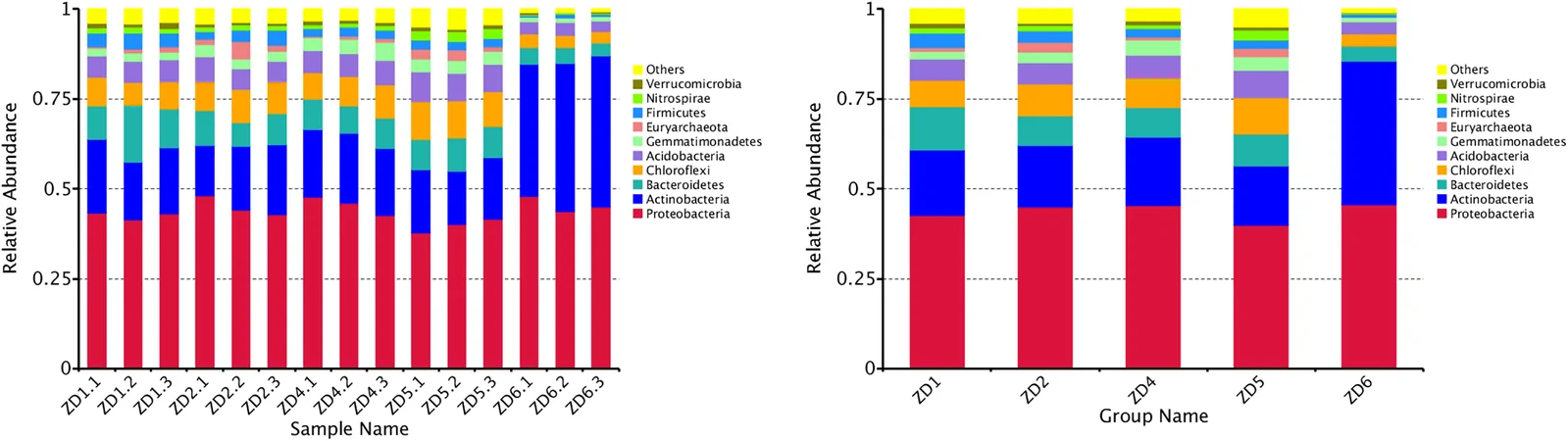
Relative abundance of bacterial phyla in the rhizosphere of *Xanthium strumarium.* The stacked bar charts show the taxonomic composition of the microbial community at the phylum level. The chart on the left displays results for individual samples, while the chart on the right shows the averaged composition for each sampling site group (ZD1-ZD6). Each color corresponds to a specific bacterial phylum as indicated in the legend. “Others” represents the sum of all phyla not included in the top ten most abundant.

**Fig 10:**
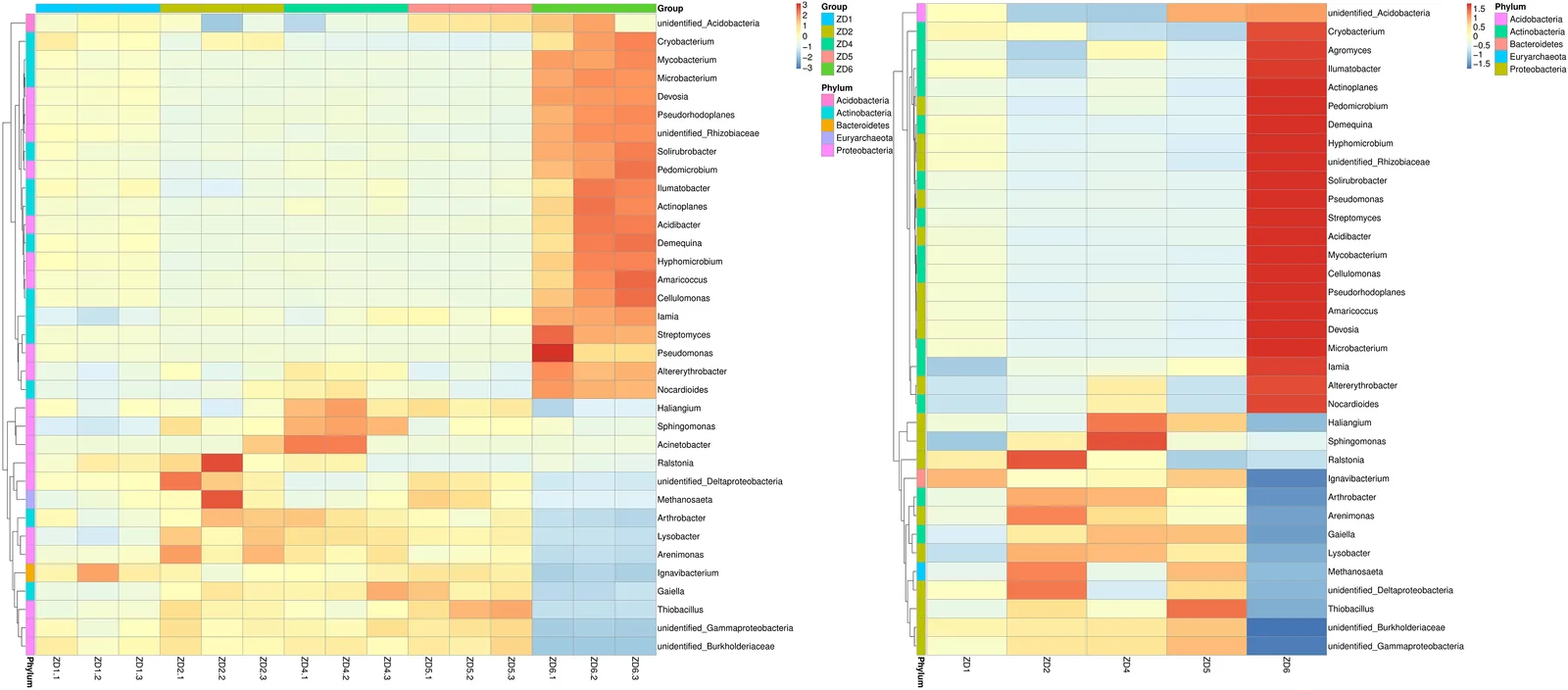
Heatmap showing the relative abundance and hierarchical clustering of bacterial genera. This figure is presented in two panels for clarity, showing different sets of genera. Rows represent individual samples, and columns represent bacterial genera. The color intensity reflects the Z-score standardized relative abundance, with red indicating higher abundance and blue indicating lower abundance. The dendrogram on the left clusters the samples based on the similarity of their microbial composition.

The structure of the rhizosphere microbial community, as illustrated by relative abundance and clustering analyses (Figs 9 and 10), exhibited differences across samples and groups. At the phylum level, Proteobacteria was the most abundant phylum in most samples, with its relative abundance often exceeding 50% in sites ZD1 and ZD2. Firmicutes and Bacteroidetes were present across all samples with a relatively even distribution, while Acidobacteria showed a higher relative abundance in samples from sites ZD2 and ZD4. When averaged by group, samples from sites ZD5 and ZD6 contained a higher proportion of taxa classified as ‘Others’. The relative abundances of Nitrospira and Verrucomicrobia were highest in the ZD6 group. At the genus level, Pseudomonas and Streptomyces were most abundant in the ZD5 group (Fig 10).

A PERMANOVA (Adonis) analysis confirmed that the observed differences in microbial community composition among the sampling sites were statistically significant. Pairwise comparisons showed that the microbial communities of ZD6 were significantly different from all other sites, particularly from ZD5 (p < 0.001; R^2^ = 0.9315) and ZD1 (p < 0.001; R^2^ = 0.8860). Significant differences were also found between sites ZD1 and ZD4 (p < 0.001; R^2^ = 0.6619), ZD1 and ZD5 (p < 0.001; R^2^ = 0.6873), and ZD1 and ZD2 (p < 0.001; R^2^ = 0.5704), indicating strong habitat-specific influences on the microbiome.

### 3.6 Functional prediction of soil microorganisms

**Fig 11:**
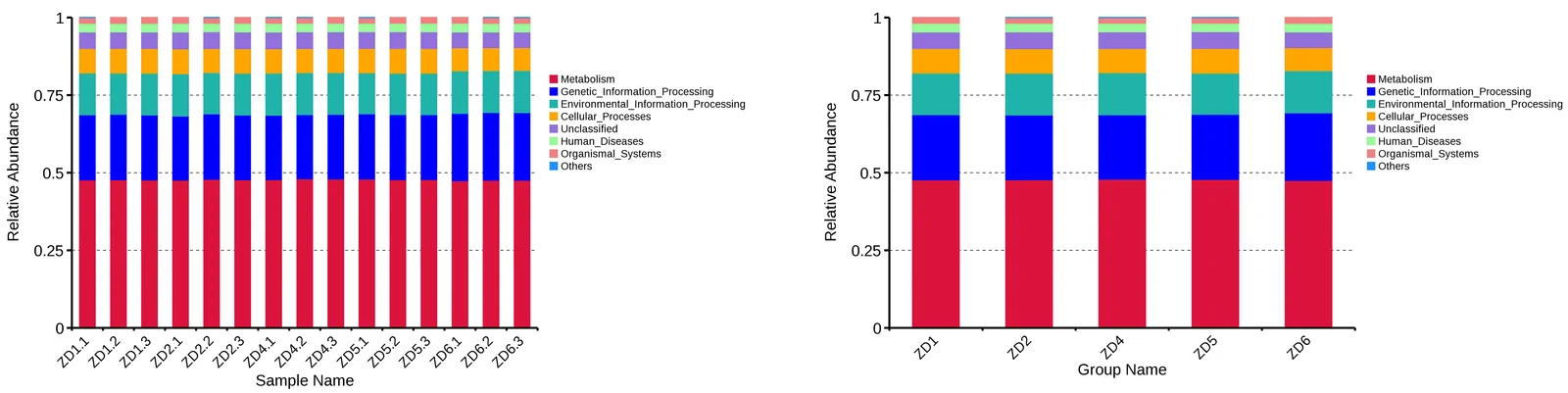
The Tax4Fun functional annotation relative abundance bar chart. The x-axis represents the sample name; the y-axis represents relative abundance; ‘Others’ represents the sum of the relative abundance of all categories beyond the top 10 categories in the chart.

**Fig 12:**
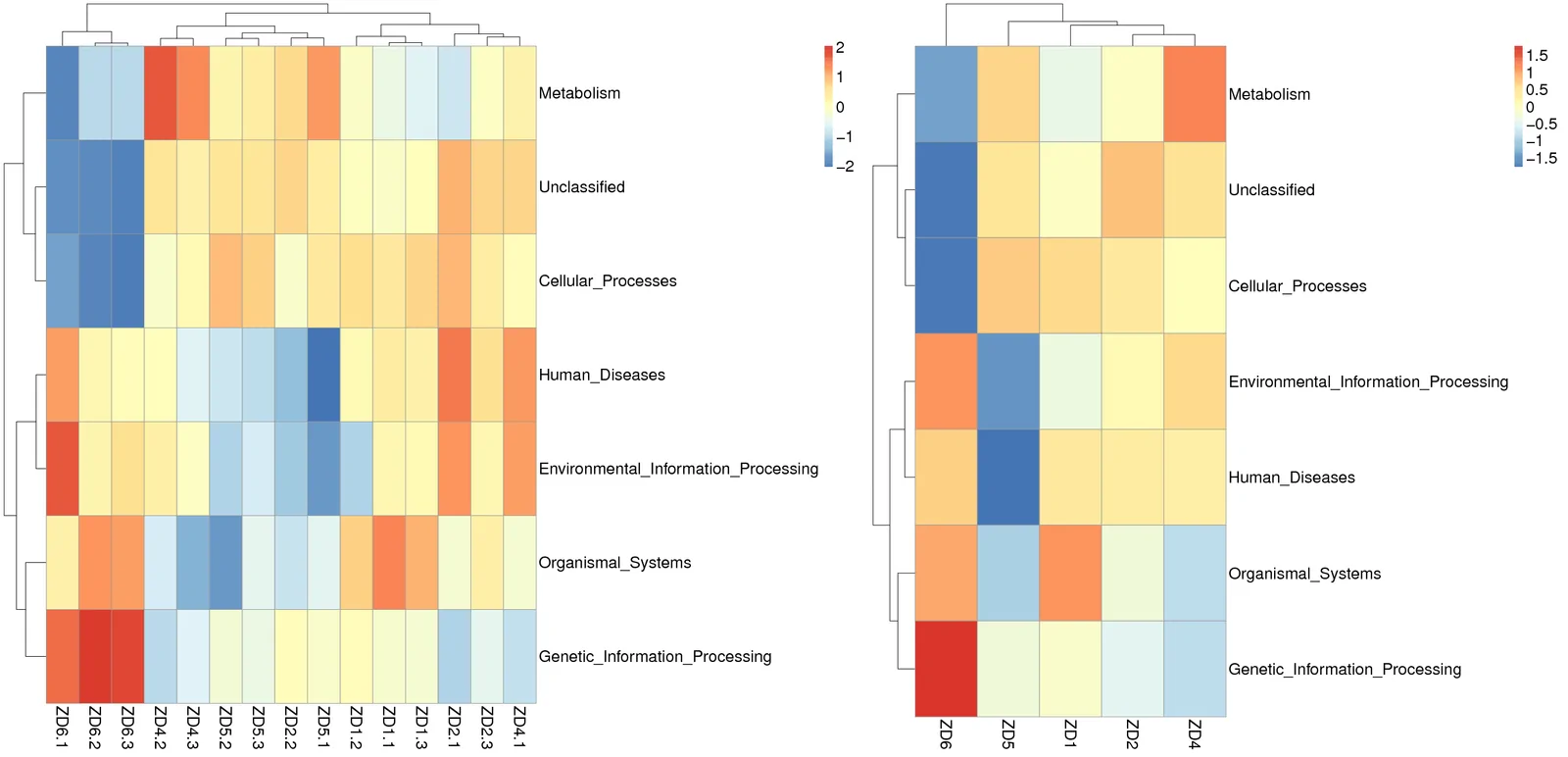
The Tax4Fun functional annotation clustering heatmap. The vertical axis represents sample information, and the horizontal axis represents species annotation information. The clustering tree on the left side of the chart is the species clustering tree. The values in the heatmap correspond to the Z-scores of the relative abundance of each species in each row after standardization. That is, the Z-score for a sample in a specific classification is the difference between the relative abundance of the sample in that classification and the average relative abundance of all samples in that classification, divided by the standard deviation of the relative abundance of all samples in that classification.

**Fig 13:**
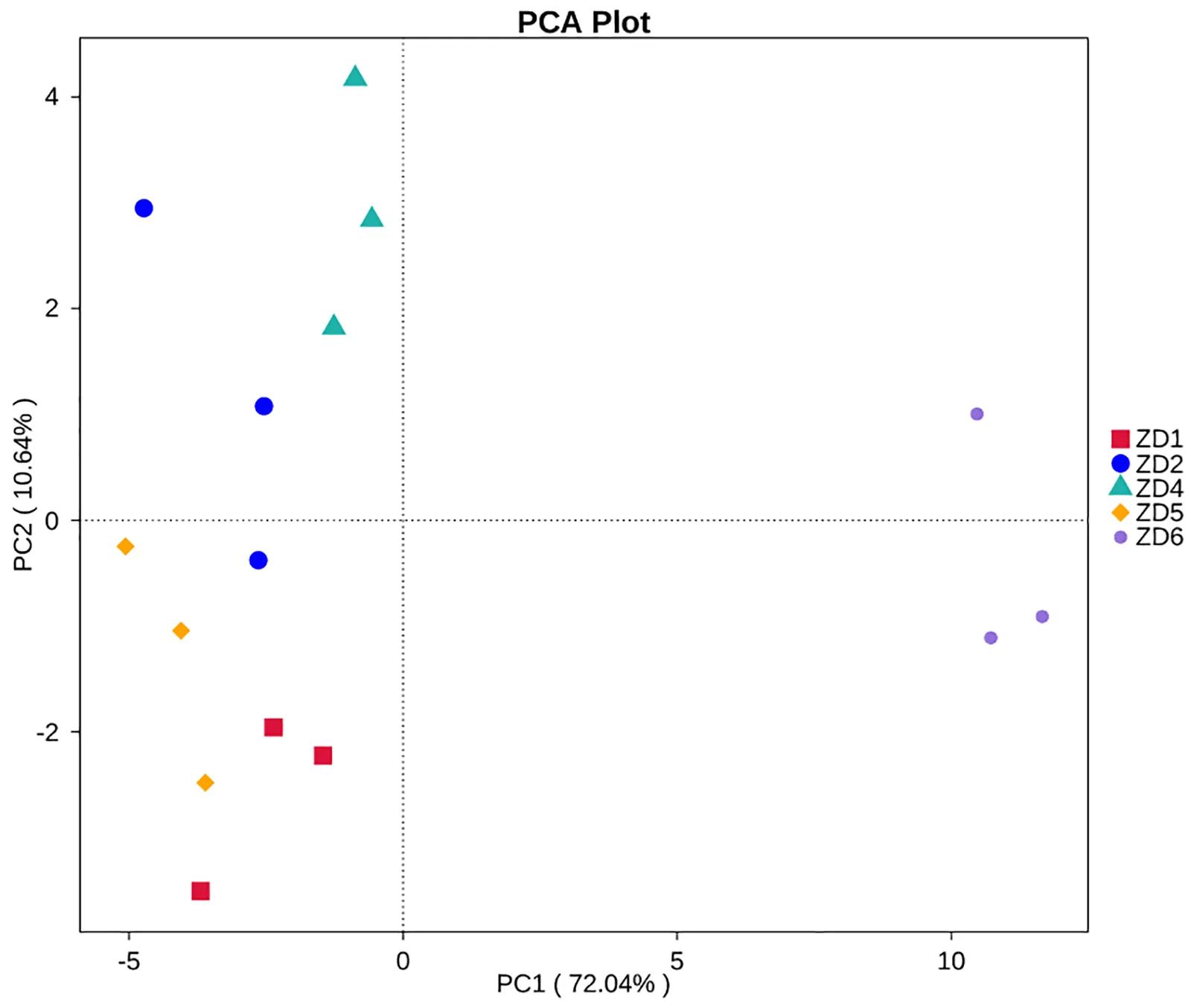
Display of Tax4Fun functional annotation PCA results. The scatter plot shows the ordination of samples based on their predicted functional profiles. Each point represents a single sample, with its shape and color corresponding to the sampling site (ZD1-ZD6) as indicated in the legend. The proximity of points reflects the similarity of their microbial functional composition. The percentage of total variance explained by the first two principal components (PC1 and PC2) is shown on the axes.

The predicted functional profiles of the rhizosphere microbial communities, based on Tax4Fun analysis, showed differences across the samples and groups (Figs 11 and 12). At KEGG Level 1, ‘Metabolism’ was the most abundant functional category in all samples, with its relative abundance generally approaching 0.5. ‘Genetic Information Processing’ was the second most abundant category. In contrast, the relative abundances of categories such as ‘Human Diseases’ and ‘Organismal Systems’ were minimal across all samples. When analyzed by group, the ZD6 group presented the highest relative abundance of ‘Metabolism’ and a comparatively lower abundance of ‘Genetic Information Processing’ and ‘Environmental Information Processing’. The Principal Component Analysis (PCA) showed that samples from the same site tended to cluster together, with the ZD6 samples forming a distinct cluster separated from the other groups along the PC1 axis (Fig 13).

## 4 Discussion

### 4.1. Impact of climate change on habitat distribution of *Xanthium strumarium*

This study applied the widely used Biomod2 model to explore the impact of global climate change on the suitable habitats of *X. strumarium* in China. Our results indicate that climate change is expected to significantly alter the distribution of suitable habitats for this species, with varying impacts depending on emission scenarios. Under current climate conditions, the primary suitable habitats for *X. strumarium* are concentrated in southern, central, eastern, northeastern, and southeastern China, representing regions with optimal climatic conditions for plant growth.

Given the substantial influence of global warming on the projected changes in precipitation and climate patterns, the expansion and contraction of plant species are expected to have strong ecological impacts across China. Under future climate scenarios, particularly those associated with high emissions (SSP5–8.5), the distribution of suitable habitats is predicted to expand, driven by rising temperatures and changing precipitation patterns. These changes are expected to create favorable conditions for the species in areas where competition was previously less intense. This expansion is further exacerbated by the increasing frequency of extreme weather events, such as floods, which create the disturbed habitats where *X. strumarium* excels at outcompeting native flora. The predicted expansion primarily includes regions such as Yunnan, Guizhou, Guangdong, Hainan, and parts of Inner Mongolia, with coastal areas also becoming increasingly susceptible to invasion.

Despite a projected increase in total habitat availability, Table 3 shows a significant reduction in high-risk zones under the SSP5–8.5 scenario. This indicates that *X. strumarium* does not respond linearly to extreme climate scenarios; instead, intensified environmental stressors may compromise the suitability of current infestation centers. As conditions drift beyond optimal thresholds, these core areas experience ‘suitability decay,’ while the species simultaneously colonizes new marginal environments. This shift necessitates a broader management perspective that prioritizes emerging risk zones as potential corridors for future migration.

Climate change can promote plant invasions by increasing disturbances through extreme climatic events and changing environmental conditions. Previous studies have identified different key environmental variables for *X. strumarium* in various regions. For example, Kekeç and Kadıoğlu [98] identified Bio4, Bio10, Bio11, and Bio12 as the dominant environmental variables in Turkey; Waheed *et al.* [14] highlighted Bio4, Bio8, and nitrogen content (soil nitrogen) as influential in Pakistan; and Chikuruwo *et al.* [99] found NDVI to be the most significant factor in southeastern Zimbabwe. The variation in these influential environmental variables across studies may be due to differences in study areas, selected variables, and distribution points [100]. In this study, we identified human activity (human footprint) as the dominant environmental variable affecting *X. strumarium* distribution, followed by topography, and then NDVI along with a series of bioclimatic variables. This aligns with our finding that human footprint is the most significant predictor, contributing 66.6% to the model's explanatory power (Fig 5), far surpassing other variables. The dominance of the human footprint index likely acts as a proxy for specific edaphic conditions favoring this species. Recent evidence emphasizes that soil chemical properties, particularly Cation Exchange Capacity (CEC) and soil available nitrogen, are critical drivers for the distribution of nitrophilous invasive species. Anthropogenic disturbances typically disrupt soil structure and increase nutrient availability, creating the high-nitrogen micro-environments required for *X. strumarium* colonization [101]. Therefore, while our broad-scale model relies on the human footprint as a macro-indicator, this variable essentially captures the species’ dependence on fertile, disturbed soil conditions characterized by altered CEC and nitrogen levels. To further refine prediction precision, particularly in agricultural interfaces, future risk assessments should explicitly integrate high-resolution raster data of these edaphic factors. Meanwhile, the changes in the dynamic ecological niche reflect a decrease in the ecological adaptability of *X. strumarium* with the increase in fossil fuel emissions. The niche dynamics analysis (Fig 8) further shows a modest decline in niche stability, with niche expansion (indicated by the red shading) occurring in new environmental spaces that the species is adapting to under these altered conditions.

In conclusion, the predicted expansion of suitable habitats under climate change, particularly in regions with increased human activity and altered topography, highlights the ongoing shifts in the potential distribution of *X. strumarium* across China.

### 4.2 Role of rhizosphere microbial communities in adaptation

The rhizosphere microbial communities play a pivotal role in the adaptation of *X. strumarium* to diverse environmental conditions. The microbial community structure varies significantly across different samples and groups, with Proteobacteria being the dominant phylum in most samples, suggesting its strong role in soil metabolism and nutrient cycling [102,103]. Other microbial phyla like Firmicutes and Bacteroidetes contribute to the adaptability of *X. strumarium* in various soil conditions, including nutrient-deficient and acidic environments [104,105]. Emerging evidence utilizing multi-omics approaches highlights that plants actively reshape their rhizosphere microbiome to select for taxa that enhance stress tolerance and resource acquisition under changing environment [106,107]. This mechanism likely facilitates the establishment of *X. strumarium* in novel ranges by recruiting beneficial nitrogen-cycling taxa. Additionally, the higher microbial diversity observed in samples such as ZD5 and ZD6, coupled with the increased proportion of Nitrospira and Verrucomicrobia, points to active nitrogen cycling in these habitats, which could help improve nutrient availability for the plant. For instance, the high abundance of Nitrospira (Fig 9) suggests robust nitrification processes, while Verrucomicrobia are often associated with the degradation of complex organic matter, both of which are critical for nutrient acquisition. Our functional prediction analysis (Fig 11) confirms that the ‘Metabolism’ pathway is the most abundant, supporting the idea that these microbial communities provide metabolic flexibility for the host plant. The interactions between the plant and its rhizosphere microbiome support its growth in disturbed or marginal soils, allowing *X. strumarium* to thrive in diverse and challenging environments.

### 4.3 Uncertainties of the present study

In this study, we first employed an integrated modeling approach to simulate the potential distribution of *X. strumarium* in China under current and future climate scenarios, thereby reducing uncertainties arising from differences in model algorithms. Although the distribution points were carefully selected, the spatial dispersion of these points and the lack of field surveys in some regions may have influenced the prediction of the species’ potential suitable areas. The selection of environmental variables plays a crucial role in species distribution prediction and model accuracy. This study did not account for various factors such as soil characteristics and land use, which could significantly impact the species’ distribution. Future research should consider incorporating additional environmental variables and conduct field surveys in other provinces to supplement the data. Furthermore, dispersal dynamics can notably influence a species’ response to global changes, potentially affecting the study’s outcomes [108,109]. Moreover, an important uncertainty in bridging macro-ecological models with micro-ecological data is the issue of spatial scale. While our rhizosphere microbial analysis provides valuable localized mechanistic insights into how *X. strumarium* adapts to high-disturbance environments along an anthropogenic gradient, our sampling was restricted to specific sites in Inner Mongolia. The lack of direct integration of nationwide microbial sampling with the predictive environmental raster data makes it difficult to definitively extrapolate these micro-level interactions to the entire projected distribution range. Future studies should aim to collect microbiome data across a broader latitudinal gradient and directly integrate high-resolution soil edaphic rasters into the SDM to further solidify the causal link between microbial shifts and nationwide range expansion. Future investigations could integrate different dispersal scenarios to further explore the potential changes in hotspots under climate and land use variations.

## 5 Conclusions

This study analyzes the potential suitable distribution changes of *X. strumarium* in China under climate change, dynamic ecological niche shifts, and the composition, abundance, and functional validation of its rhizosphere microbiota. The results indicate that human footprint, slope, and elevation are the dominant environmental variables influencing the distribution of *X. strumarium*. With the intensification of climate change, the potential suitable distribution of *X. strumarium* gradually expands. Moreover, the dynamic ecological niche analysis reveals a reduction in the ecological adaptability of *X. strumarium* with the increasing fossil fuel emissions. The validation of rhizosphere microbiota abundance and function provides localized mechanistic insights into how *X. strumarium* tolerates disturbance and adapts to emerging environments, corroborating its ecological resilience. This study provides insights into the potential distribution changes, dynamic ecological niche shifts, and the composition and function of soil rhizosphere microbiota of *X. strumarium* under climate change, offering theoretical support for the future utilization and management of *X. strumarium*.
