# Supplementary material for: Climate change impacts on Xanthium strumarium distribution: Integrating species distribution models with rhizosphere microbiome analysis in China
Source: PLoS One. 2026 Aug 21;21(8):e0351471. doi: 10.1371/journal.pone.0351471 (PMC13496439; doi:10.1371/journal.pone.0351471)
Supplement: Supplementary file 1 [file pone.0351471.s001.docx]

**Introduction**

1. Why authors have chosen Biomod2 over other alternative modeling frameworks. Justify it.
2. Authors should abbreviate species names after the first mention. For example, it is acceptable to use *Xanthium strumarium* the first time, but in subsequent mentions, the abbreviated form *X. strumarium* should be used consistently throughout the manuscript
3. The dual role of *X. strumarium* is mentioned in the introduction; however, the specific challenges it poses in China—such as its impact on agriculture—are not sufficiently detailed. Additionally, while its medicinal value is briefly referenced, this aspect could be further elaborated to enhance the context and significance of the study.

**Material and methods**

1. Line 108, Provide rationale why resampling to 5 km was necessary.
2. Table 1, why specific environmental variables such as slope and NDVI were chosen for the analysis. Clarify, how these are relavant to *Xanthium strumarium*.
3. Ensure that all figures and tables are cited consistently and placed logically within the text. Notably, Figure 1 is not mentioned or referenced in the manuscript
4. Line 143, Briefly justify the selection of the 12 algorithms and list all 12 algorithms by their full name and acronym.
5. Clarify the absence of Table 2 in the manuscript and address the duplication of Table 3. Ensure that all tables are correctly numbered, referenced in the text, and presented in a logical sequence.
6. Authors should consider about adding a methodological flow diagram for better understanding.

**Results**

1. Line 189, Define the full names of acronyms when they first appear to ensure clarity. (e.g., FDA, MARS)
2. Line 213, The choice of the EMmean method is mentioned in the manuscript but not explained. Justify why this method is preferred over other methods.
3. Figure 3 includes AUC, Kappa, and TSS values. While AUC and Kappa are discussed in detail, TSS is not addressed in the results interpretation. Please either include a comprehensive discussion of the TSS results or clarify the reason for its omission to maintain consistency and completeness in the evaluation metrics.
4. Some figures are not self-explanatory. Captions are brief and lack units or explanations. (e.g., Make sure that colour gradient in figure 5 is clearly explained in the caption)
5. Provide a brief description about figure 5 and 6 in the text.
6. Legend of Figure 7 is missing.
7. The results lack statistical tests to validate differences between microbial communities or habitat prediction in section 3.5. Use ANOVA/PERMANOVA or any other method to statistically compare microbial diversity and habitat suitability across groups and scenarios.

**Discussion**

1. Discuss about the role of extreme weather events (e.g., floods) in shaping the distribution and competitive dynamics of X. strumarium.
2. Human activity is identified as the dominant environmental variable influencing *X. strumarium*, but its role is not adequately explained.
3. “Role of Rhizosphere Microbial Communities in Adaptation” section of the discussion could be improved by addressing how microbial interactions may shift in response to climate change.
4. While the role of microbial communities in adaptation is highlighted, the mechanisms by which specific microbial phyla contribute to plant adaptation remain underexplored. Elaborate on the functional roles of key microbial groups like *Verrucomicrobia* and *Nitrospira*.
5. Figures and quantitative data are not sufficiently integrated into the discussion.

**General Comments**

1. Split Results and Discussion clearly. Some interpretation slips into Results.
2. Several sentences are grammatically incorrect; a thorough proofreading is recommended to improve the clarity.
3. Figures are not self-explanatory. Legends are brief, kindly expands it. Also increase the resolution of the figures.
4. Species names must be italicized throughout the manuscript.
