# Supplementary material for: Climate change impacts on Xanthium strumarium distribution: Integrating species distribution models with rhizosphere microbiome analysis in China
Source: PLoS One. 2026 Aug 21;21(8):e0351471. doi: 10.1371/journal.pone.0351471 (PMC13496439; doi:10.1371/journal.pone.0351471)
Supplement: Supplementary file 2 [file pone.0351471.s003.docx]

The manuscript was written well but some modifications are required

Xanthium strumarium, an invasive species which can disrupt biodiversity/ecosystems, reduce crop yields, and degrade pastures, leading to economic losses. To address the challenges posed by invasive species the investigation was undertaken to find out potential distribution and how it might respond to climate change. In the abstract part, potential risk zones may be included for better understanding.

It was observed that to investigate the potential impacts of climate change on the distribution of *X. strumarium*, species distribution model was used. It may be more better if the model predict No Risk Zones, Low-Risk Zones , Medium Risk Zones, High-Risk Zones etc

During the analysis of distribution pattern, soil chemical properties like CEC, soil available N etc maybe include.

Discussion part maybe enriched with more recent evidences as well as recent references.
