# Supplementary material for: Climate change impacts on Xanthium strumarium distribution: Integrating species distribution models with rhizosphere microbiome analysis in China
Source: PLoS One. 2026 Aug 21;21(8):e0351471. doi: 10.1371/journal.pone.0351471 (PMC13496439; doi:10.1371/journal.pone.0351471)
Supplement: Supplementary file 3 [file pone.0351471.s004.docx]

Response to Reviewers

Manuscript ID: PONE-D-25-30270

Title: Climate Change Impacts on *Xanthium strumarium* Distribution: Integrating Species Distribution Models with Rhizosphere Microbiome Analysis in China

Dear Academic Editor and Reviewers,

Thank you for your valuable feedback and thoughtful comments on our manuscript, which have helped us to significantly improve its quality. We have carefully addressed each point raised and have revised the manuscript accordingly. Our point-by-point responses are detailed below. All changes made in the manuscript are highlighted with track changes in the revised file.

**Part A: Responses to Journal Requirements**

We have carefully reviewed and revised the manuscript to comply with PLOS ONE's style requirements, including those for formatting and file naming. We have added a statement in the “Materials and Methods” section (Section 2.3) to clarify that no specific permits were required for the field sampling. The soil samples were collected from public-access areas in Inner Mongolia, China, and did not involve any protected species or private lands.

The revised statement is: “The Inner Mongolia Autonomous Region Natural Science Foundation Project (2023LHMS03007) provided funding for the article processing charges (APC) of this publication. The funder had no role in the study design, data collection and analysis, decision to publish, or preparation of the manuscript.”

We confirm that all relevant data necessary to replicate the study's findings are included within the manuscript and its Supporting Information files.

The corresponding author's ORCID iD has been created and validated in the Editorial Manager system.

We apologize for the oversight regarding the copyrighted maps. We confirm that Figures 1, 6, and 7 (the originally referenced Fig 5 and 6) were produced using standard map data from the official standard map service of the Ministry of Natural Resources of China, which are publicly available for scientific use. We have added a specific statement to the captions of these figures to reflect this, including the official map approval number GS(2023)2767. This demonstrates that the base maps are officially approved and publicly accessible. The revised captions for these figures are as follows:

Fig 1: An integrated methodological framework for analyzing climate change impacts on *Xanthium strumarium* distribution and its rhizosphere microbiome. The geographical components within this figure are based on standard map data from the official standard map service of the Ministry of Natural Resources of China (map approval number GS(2023)2767), used without modification.

Fig 6: Predicted potential distribution of suitable habitats for *Xanthium strumarium* in China under current environmental conditions. The base map for this figure was generated using standard map data from the official standard map service of the Ministry of Natural Resources of China (map approval number GS(2023)2767), with the geographical elements used without modification.

Fig 7: Projected future distributions of suitable habitats for *Xanthium strumarium* in China. The base map for this figure was generated using standard map data from the official standard map service of the Ministry of Natural Resources of China (map approval number GS(2023)2767), with the geographical elements used without modification.

We have revised the manuscript to ensure that Figure 1 is correctly cited and placed logically within the text in the “Materials and Methods” section. We have also added a detailed caption for Figure 7, which was previously missing. We apologize for the confusion and have now added the missing Table 2 (Rhizosphere soil sampling sites) to the manuscript, and all subsequent tables have been renumbered to ensure a logical sequence. No specific citations were recommended by the reviewer, so no action was required here.

**Part B: Responses to Reviewer Comments**

Reviewer #1

Introduction

Justify Biomod2 selection. We have revised the Introduction to provide a clear justification for choosing the Biomod2 framework. We highlight its ability to integrate multiple modeling algorithms, which reduces the uncertainty inherent in single models and provides a more robust and comprehensive prediction.

Abbreviate species names. We have thoroughly reviewed the manuscript and have consistently abbreviated *Xanthium strumarium* to *X. strumarium* after its first mention in the abstract and introduction.

Elaborate on the dual role of *X. strumarium*. We have expanded the Introduction to provide more detailed context on the dual nature of *X. strumarium*. We have added specific examples of its negative impact on Chinese agriculture, such as its invasion of farmlands and contamination of crops and livestock products, to enhance the significance of the study.

Materials and Methods

Rationale for 5 km resampling. We have added a sentence in the “Data Sources and Processing” section to explain the rationale for selecting a 5 km resolution. This scale was chosen to ensure methodological consistency, ecological appropriateness for a widespread species, and to minimize noise and spatial inaccuracies in the occurrence data.

Justify selection of variables. We have clarified the relevance of specific environmental variables, such as slope and NDVI, to *X. strumarium* in the “Data Sources and Processing” section. We explain that these variables are ecologically relevant to a ruderal species that thrives in disturbed and low-gradient environments.

Consistent figure and table citations. We have corrected all figure and table citations to ensure they are consistent and logically placed within the text.

Justify and list 12 algorithms. We have corrected the number of algorithms from 12 to 11, as Support Vector Machine (SVM) was initially part of the framework but was excluded. We apologize for this error. We have updated the text in “Models Construction and Changes in Ecological Niches” to clearly state that 11 algorithms were used. The full names and acronyms of all 11 algorithms are already listed in the manuscript. We have also added a brief justification for using an ensemble of diverse algorithms, explaining that this approach captures a wide range of species-environment relationships and reduces uncertainty.

Clarify table numbering. We apologize for the confusion. We have added the missing Table 2 (Rhizosphere soil sampling sites) to the manuscript, and all subsequent tables have been renumbered to ensure a logical sequence.

Add a methodological flow diagram. We have added a methodological flow diagram (Fig 1) to the “Materials and Methods” section, which visually summarizes the entire research process.

Results

Define acronyms. The full names of all acronyms, including FDA and MARS, are defined in the text when they first appear. We have reviewed the manuscript to ensure this consistency.

Justify the EMmean method. As requested in the provided document, we have already added a comprehensive justification for the choice of the EMmean method, highlighting its robustness, transparency, and ability to balance predictions from diverse algorithms.

Discuss TSS values. We have added a sentence to the “Model Precision Assessment” section to briefly interpret the TSS values, mentioning that the RF model achieved the highest score and that several other models also performed well, providing a consistent analysis of all three metrics (AUC, TSS, and Kappa).

Improve figure captions. We have expanded the captions for Figures 4, 5, 6, 7, 8, 9, 10, 11, 12, and 13 to be more self-explanatory. We have also added units and clearer explanations for color gradients and other visual elements, ensuring readers can understand the figures without referring to the main text.

Provide a brief description about figures 5 and 6 in the text. We have revised the “Bioclimatic Variable Contribution” and “Present Potential Geographic Spread” sections to include brief descriptions and references to Figures 5 and 6, respectively.

Legend for Figure 7. We have added a clear and descriptive legend for Figure 7.

Add statistical tests for microbial communities. We apologize for this oversight. We have now performed a PERMANOVA (Adonis) analysis to statistically compare the microbial community composition among the different sampling sites. We have added the results of this analysis to the “Rhizosphere Microbial Abundance” section (Section 3.5), including the p-values and R² values, which now statistically validate the observed differences.

Discussion

Role of extreme weather events. We have added a sentence to the “Impact of Climate Change on Habitat Distribution” section to discuss the role of extreme weather events, such as floods, in creating disturbed habitats that promote the spread and competitive advantage of *X. strumarium*.

Clarify the role of human activity. We have expanded the discussion on the dominant role of the human footprint. We now explicitly state that the human footprint index captures the cumulative impact of land-use changes like agricultural expansion and urbanization, which create the disturbed environments where this species thrives. We have also integrated the quantitative data from Fig 5 to highlight its significant contribution (66.6%) to the model.

Microbial interactions and climate change. We have added a sentence to the “Role of Rhizosphere Microbial Communities in Adaptation” section to discuss how microbial interactions may shift in response to climate change, specifically mentioning that altered soil moisture and temperature could lead to an increase in stress-tolerant microbial taxa.

Functional roles of Verrucomicrobia and Nitrospira. We have added a detailed explanation of the functional roles of Verrucomicrobia and Nitrospira, clarifying their contributions to nitrogen cycling and the degradation of complex organic matter. We also linked these functions to the overall 'Metabolism' pathway predicted by Tax4Fun to provide a deeper mechanistic insight.

Integrate figures and data. We have thoroughly reviewed the Discussion section and have integrated quantitative data and direct references to Figures 5, 8, 9, and 11 to support our interpretations and conclusions, ensuring the discussion is well-supported by the results.

General Comments

Split Results and Discussion. We have re-evaluated the manuscript and have split the “Results” and “Discussion” sections more clearly. We have moved all interpretive sentences to the Discussion and ensured that the Results section focuses solely on presenting the data and findings.

Proofreading. The manuscript has undergone a thorough proofreading to correct grammatical errors and improve the overall clarity and readability.

Figure clarity and resolution. As stated in our response to Reviewer 1's comments on the Results section, we have expanded all figure captions to make them more self-explanatory. We have also ensured that all figures meet the specified resolution requirements for publication.

Italicize species names. We have performed a final check to ensure that all species names (*Xanthium strumarium* and *X. strumarium*) are consistently italicized throughout the manuscript.

We believe that these revisions have significantly improved the quality and rigor of our manuscript. We are confident that it now meets the publication criteria for PLOS ONE. We look forward to your positive decision.

Sincerely,

Jinyang Dong & Liyan Zhang

Co-first authors
