# Supplementary material for: Climate change impacts on Xanthium strumarium distribution: Integrating species distribution models with rhizosphere microbiome analysis in China
Source: PLoS One. 2026 Aug 21;21(8):e0351471. doi: 10.1371/journal.pone.0351471 (PMC13496439; doi:10.1371/journal.pone.0351471)
Supplement: Supplementary file 4 [file pone.0351471.s005.docx]

Manuscript ID: PONE-D-25-30270R1

Title: Climate Change Impacts on Xanthium strumarium Distribution: Integrating Species Distribution Models with Rhizosphere Microbiome Analysis in China

Authors: Jinyang Dong, Liyan Zhang, Xiaoli Wang, Yanru Zhang, Mengzhe Zhu, and Haiyan Jiang

To: Dr. Debasis Mitra Academic Editor, PLOS ONE

Dear Dr. Mitra,

Thank you very much for giving us the opportunity to revise our manuscript. We sincerely appreciate the time and effort you and the reviewers have dedicated to providing insightful feedback on our work.

We have carefully considered the comments raised by Reviewer #3 (as contained in the attachment). We found the suggestions extremely constructive and have addressed each point in detail. The manuscript has been revised to incorporate invasion risk zoning, discussions on soil chemical properties, and updated literature from 2023–2025.

Below is a point-by-point response to the specific comments.

Response to Reviewer #3

Comment 1: "The manuscript was written well but some modifications are required. *Xanthium strumarium*, an invasive species... In the abstract part, potential risk zones may be included for better understanding."

Response: We appreciate this suggestion. We have revised the Abstract to explicitly identify high-risk areas. The text now reads: "Future climate scenarios predict habitat expansion of 8.9–28.6%, identifying high-risk invasion zones primarily concentrated in Yunnan, Guangdong, and Inner Mongolia provinces." This modification provides a clearer warning for management purposes.

Comment 2: "It was observed that to investigate the potential impacts of climate change on the distribution of *X. strumarium*, species distribution model was used. It may be more better if the model predict No Risk Zones, Low-Risk Zones, Medium Risk Zones, High-Risk Zones etc."

Response: We fully agree that classifying results into risk zones is more practical for invasion management.

Methodology Update: In Section 2.2, we reclassified the habitat suitability indices into four discrete categories: No-Risk Zone (0–0.25), Low-Risk Zone (0.25–0.50), Medium-Risk Zone (0.50–0.75), and High-Risk Zone (0.75–1.0).

Visuals Updated: We have updated Figure 6, Figure 7, and Table 3 to reflect this new terminology.

Comment 3: "During the analysis of distribution pattern, soil chemical properties like CEC, soil available N etc maybe include."

Response: This is a critical point. While our broad-scale SDM relied on climatic and topographic variables due to the limitations of high-resolution soil raster data at the national scale, we have significantly strengthened the discussion on soil chemical properties by linking them to our Human Footprint and Microbiome results.

In Section 4.1: We expanded the discussion to explain that the high contribution of the "Human Footprint" variable serves as a proxy for nutrient-rich soil conditions caused by anthropogenic disturbance. We explicitly discussed how CEC and Soil Available Nitrogen act as drivers for this nitrophilous species, citing recent evidence.

In Section 4.2: We utilized our molecular data to provide biological validation that soil nitrogen cycling is indeed a key limiting factor for *X. strumarium*, effectively bridging the gap between macro-models and micro-soil properties.

Comment 4: "Discussion part maybe enriched with more recent evidences as well as recent references."

Response: We have extensively updated the Discussion section with literature published between 2023 and 2025 to ensure the manuscript reflects the state-of-the-art.

Mechanisms of Invasion: We incorporated recent studies on how disturbance releases nutrients.

Microbiome Adaptation: We added references regarding how invasive plants actively reshape microbial communitie.

Soil Heterogeneity: We cited recent findings on soil nutrient heterogeneity.

Journal Requirements regarding References: We have reviewed our reference list to ensure completeness and correctness. We confirm that no retracted papers have been cited.

We believe that these revisions have significantly improved the quality and clarity of the manuscript.

Sincerely,

Dr. Haiyan Jiang (Corresponding Author)

Department of Forest Protection, College of Forestry Inner Mongolia Agricultural University

Email: jhydlm@126.com
