# Supplementary material for: Climate change impacts on Xanthium strumarium distribution: Integrating species distribution models with rhizosphere microbiome analysis in China
Source: PLoS One. 2026 Aug 21;21(8):e0351471. doi: 10.1371/journal.pone.0351471 (PMC13496439; doi:10.1371/journal.pone.0351471)
Supplement: Supplementary file 5 [file pone.0351471.s006.docx]

Dear Editor,

Thank you for giving us the opportunity to submit a revised draft of our manuscript. We deeply appreciate the time and effort that you and the reviewers have dedicated to providing valuable, constructive, and insightful feedback. We have carefully read all the comments and found them immensely helpful in improving the quality, logical rigor, and clarity of our study.

In this revised manuscript, we have addressed all the concerns raised by the reviewers. Specifically, the most significant improvements include:

Clarifying the Spatial Logic: We have explicitly detailed the rationale for selecting the five microbial sampling sites in Inner Mongolia, seamlessly bridging our macro-scale Species Distribution Model (SDM) predictions (specifically the Human Footprint Index) with our micro-scale microbiome findings.

Explaining Ecological Trends: We have provided a robust ecological interpretation for the projected decrease in the high-risk zones of *Xanthium strumarium* under future high-emission scenarios (the "suitability decay" phenomenon).

Refining Conclusions: We have carefully calibrated our language to acknowledge the spatial limitations of the microbial sampling, presenting the findings as vital mechanistic insights rather than definitive nationwide proofs.

Following this summary, a detailed, point-by-point response to each of the comments raised by the editor and reviewers is presented below.

Comment 1: The reasons for the decrease, instead of increase, in the areas of high-risk zones as shown in Table 7.

Response: We deeply appreciate the reviewer's rigorous review and insightful observation. First, we would like to gently clarify that the projected changes in the distribution areas, including the high-risk zones, are detailed in Table 3 and Figure 7 of our manuscript (we respectfully assume the mention of Table 7 was a minor typographical oversight).

The decrease in high-risk zones under future scenarios—particularly under the high-emission SSP5-8.5 scenario—is indeed a counterintuitive but ecologically plausible phenomenon, rather than a model artifact. This non-linear trend, which we refer to as "suitability decay," can be attributed to the following key factors:

Physiological Thresholds: Under extreme warming scenarios, temperature and moisture stress in the current core habitats may exceed the optimal physiological limits for *X. strumarium.* As environmental conditions drift beyond these optimal thresholds, the habitat quality in these historic core areas degrades from "High-Risk" down to "Medium- or Low-Risk."

Niche Fragmentation and Range Shift: While the overall total suitable area expands (specifically, driven by a significant increase in Low- and Medium-Risk zones as shown in Table 3), the highly concentrated optimal "hotspots" undergo spatial fragmentation. The species is simultaneously colonizing new, marginal environments at higher latitudes or altitudes, which initially present as lower-risk zones.

Environmental Trade-offs: Our model indicates that the Human Footprint Index is the dominant driver (contributing 66.6%). While anthropogenic disturbance provides the necessary fertile micro-environments, the intensifying macro-climatic stressors in future scenarios counterbalance these benefits. This trade-off reduces the overall probability of maintaining "optimal" (high-risk) suitability in the original infestation centers.

Action taken: To ensure this phenomenon is clearly understood by readers, we have explicitly expanded upon this "suitability decay" and its implications for broader invasion management in the revised Discussion (Section 4.1).

Comment 2: Potential mirroring issues in the niche stability results under the SSP5-8.5 scenario (as shown in Figure 8).

Response: We respectfully disagree with the reviewer’s interpretation regarding a "mirroring issue." We would like to clarify that the asymmetrical protrusion observed in the bottom-left corner of the PCA space under the SSP5-8.5 scenarios (both 2041–2060 and 2061–2080) is not a graphical glitch, code error, or mirroring artifact. In the niche analysis performed via the ecospat package, the solid contours (red and green lines) delineate the outer boundaries of the available background climate space (environmental envelope) for the respective periods. The specific shape and extension of these lines are driven entirely by the underlying climate data:

Real Climate Shifts under Extreme Scenarios: The SSP5-8.5 is an extreme, high-emission scenario characterized by severe climate anomalies. The distinct "tail" or loop protruding into the negative PC1 and PC2 space explicitly reflects a dramatic shift and expansion of the macro-climatic baseline (e.g., extreme temperature/precipitation combinations or the emergence of non-analog novel climates) that occurs only under severe warming.

Evidence from Mild Scenarios: As shown in Figure 8, this protrusion is completely absent in the milder scenarios (SSP1-2.6 and SSP2-4.5), where the background climate space remains stable and symmetrical. If this were a computational or mirroring bug within the plotting script, it would consistently manifest across all climate scenarios. Its exclusive appearance under SSP5-8.5 confirms that the model is accurately visualizing the dramatic distortion of the future environmental space.

Action taken: To prevent any future misinterpretation by readers, we have added a clarifying statement in the Figure 8 caption and the corresponding Results section to explicitly state that these extended contours represent the expansion of extreme background climate spaces under the high-emission scenario.
Comment 3: The selection of microbial sampling sites—specifically, why these five locations were chosen? The lack of connection between the sites and the predictive model, and the absence of integration with environmental factors in the analysis process, make it difficult to support the conclusions about the changes in species distribution.

Response: We sincerely appreciate the reviewer’s critical and insightful comment. We agree that bridging the gap between macro-scale predictive models (SDM) and micro-scale ecological data (microbiome) requires a highly explicit logical connection. We have thoroughly revised our manuscript to clarify the strategic rationale behind our site selection and to better integrate environmental factors into our interpretation.

We have addressed this concern from the following three dimensions:

1. Strategic Site Selection Driven by SDM Predictions: The choice of Inner Mongolia and these five specific habitats was not arbitrary, but rather a targeted validation of our preliminary SDM results. First, our Biomod2 model identified Inner Mongolia as a highly vulnerable, emerging hotspot for Xanthium strumarium invasion under future climate change scenarios. Sampling at the invasion front allows us to capture the early adaptive mechanisms of expanding populations.

2. Establishing the Link: Anthropogenic Disturbance Gradient: The predictive model revealed that the Human Footprint Index (HFI) is the single most dominant macro-environmental driver of X. strumarium distribution, contributing 66.6% to the model. To directly connect our field sampling with this key environmental factor, we deliberately selected five habitats that represent a clear gradient of anthropogenic disturbance: Urban Green Space and Farmland Edge (High Disturbance) vs. Grassland, Montane Forest, and Temperate Steppe (Low to Moderate Disturbance). This allows us to investigate how macro-environmental filtering (human activities) translates into micro-ecological responses.

3. Integration of Environmental Factors and Mechanistic Insights: In the revised Discussion, we have deeply integrated these macro-environmental factors with the microbial functions. Anthropogenic disturbances typically lead to localized soil nutrient shifts, particularly nitrogen accumulation. Our microbiome data provides a direct mechanistic explanation for the SDM: in high-disturbance sites (high HFI), X. strumarium exhibits a distinct rhizosphere assembly, significantly enriching nitrogen-cycling and stress-tolerant taxa (such as Nitrospira and Pseudomonas). This micro-level microbial recruitment provides the physiological support needed for the plant to thrive in the highly suitable habitats predicted by the macro-model.

4. Calibrating the Scope of Our Conclusions: We acknowledge the spatial scale limitation raised by the reviewer—five localized sampling sites cannot fully represent nationwide species distribution shifts. Therefore, we have carefully toned down our conclusions. In the revised text, we clarify that the microbiome data serves as a localized mechanistic insight into how the species adapts to disturbance regimes, rather than a direct nationwide proof. We have also explicitly added this spatial scale mismatch as an inherent limitation in the "Uncertainties" section (Section 4.3).
