# Supplementary material for: Climate change impacts on Xanthium strumarium distribution: Integrating species distribution models with rhizosphere microbiome analysis in China
Source: PLoS One. 2026 Aug 21;21(8):e0351471. doi: 10.1371/journal.pone.0351471 (PMC13496439; doi:10.1371/journal.pone.0351471)
Supplement: Peer review history

# Peer review: Decision Letter 0

**DOI:** 10.1371/journal.pone.0351471.r001

**Authors:** Debasis Mitra

1 Aug 2025

Climate Change Impacts on Xanthium strumarium Distribution: Integrating Species Distribution Models with Rhizosphere Microbiome Analysis in China

PLOS ONE

Dear Dr. Jiang,

Thank you for submitting your manuscript to PLOS ONE. After careful consideration, we feel that it has merit but does not fully meet PLOS ONE’s publication criteria as it currently stands. Therefore, we invite you to submit a revised version of the manuscript that addresses the points raised during the review process.

Please submit your revised manuscript by Sep 15 2025 11:59PM. If you will need more time than this to complete your revisions, please reply to this message or contact the journal office at plosone@plos.org. When you're ready to submit your revision, log on to https://www.editorialmanager.com/pone/ and select the 'Submissions Needing Revision' folder to locate your manuscript file.

Please include the following items when submitting your revised manuscript:

- A rebuttal letter that responds to each point raised by the academic editor and reviewer(s). You should upload this letter as a separate file labeled 'Response to Reviewers'.
- A marked-up copy of your manuscript that highlights changes made to the original version. You should upload this as a separate file labeled 'Revised Manuscript with Track Changes'.
- An unmarked version of your revised paper without tracked changes. You should upload this as a separate file labeled 'Manuscript'.

If you would like to make changes to your financial disclosure, please include your updated statement in your cover letter. Guidelines for resubmitting your figure files are available below the reviewer comments at the end of this letter.

If applicable, we recommend that you deposit your laboratory protocols in protocols.io to enhance the reproducibility of your results. Protocols.io assigns your protocol its own identifier (DOI) so that it can be cited independently in the future. For instructions see: https://journals.plos.org/plosone/s/submission-guidelines#loc-laboratory-protocols. Additionally, PLOS ONE offers an option for publishing peer-reviewed Lab Protocol articles, which describe protocols hosted on protocols.io. Read more information on sharing protocols at https://plos.org/protocols?utm_medium=editorial-email&utm_source=authorletters&utm_campaign=protocols.

We look forward to receiving your revised manuscript.

Kind regards,

Debasis Mitra

Academic Editor

PLOS ONE

Journal Requirements:

When submitting your revision, we need you to address these additional requirements.

1.Please ensure that your manuscript meets PLOS ONE's style requirements, including those for file naming. The PLOS ONE style templates can be found at

https://journals.plos.org/plosone/s/file?id=wjVg/PLOSOne_formatting_sample_main_body.pdf and

https://journals.plos.org/plosone/s/file?id=ba62/PLOSOne_formatting_sample_title_authors_affiliations.pdf

2. In your Methods section, please provide additional information regarding the permits you obtained for the work. Please ensure you have included the full name of the authority that approved the field site access and, if no permits were required, a brief statement explaining why.

3. Thank you for stating the following financial disclosure:

“Inner Mongolia Autonomous Region Natural Science Foundation Project:

Role and Application Research of Small Secreted Proteins in Pathogenic-Symbiotic Transition Interactions (2023LHMS03007)”

Please state what role the funders took in the study.  If the funders had no role, please state: 'The funders had no role in study design, data collection and analysis, decision to publish, or preparation of the manuscript.'

If this statement is not correct you must amend it as needed.

Please include this amended Role of Funder statement in your cover letter; we will change the online submission form on your behalf.

4. We note that your Data Availability Statement is currently as follows: All relevant data are within the manuscript and in Supporting Information files.

Please confirm at this time whether or not your submission contains all raw data required to replicate the results of your study. Authors must share the “minimal data set” for their submission. PLOS defines the minimal data set to consist of the data required to replicate all study findings reported in the article, as well as related metadata and methods (https://journals.plos.org/plosone/s/data-availability#loc-minimal-data-set-definition).

For example, authors should submit the following data:

- The values behind the means, standard deviations and other measures reported;

- The values used to build graphs;

- The points extracted from images for analysis.

Authors do not need to submit their entire data set if only a portion of the data was used in the reported study.

If your submission does not contain these data, please either upload them as Supporting Information files or deposit them to a stable, public repository and provide us with the relevant URLs, DOIs, or accession numbers. For a list of recommended repositories, please see https://journals.plos.org/plosone/s/recommended-repositories.

If there are ethical or legal restrictions on sharing a de-identified data set, please explain them in detail (e.g., data contain potentially sensitive information, data are owned by a third-party organization, etc.) and who has imposed them (e.g., an ethics committee). Please also provide contact information for a data access committee, ethics committee, or other institutional body to which data requests may be sent. If data are owned by a third party, please indicate how others may request data access.

5. PLOS requires an ORCID iD for the corresponding author in Editorial Manager on papers submitted after December 6th, 2016. Please ensure that you have an ORCID iD and that it is validated in Editorial Manager. To do this, go to ‘Update my Information’ (in the upper left-hand corner of the main menu), and click on the Fetch/Validate link next to the ORCID field. This will take you to the ORCID site and allow you to create a new iD or authenticate a pre-existing iD in Editorial Manager.

6. We note that Figures 1,5 and 6 in your submission contain map/satellite images which may be copyrighted. All PLOS content is published under the Creative Commons Attribution License (CC BY 4.0), which means that the manuscript, images, and Supporting Information files will be freely available online, and any third party is permitted to access, download, copy, distribute, and use these materials in any way, even commercially, with proper attribution. For these reasons, we cannot publish previously copyrighted maps or satellite images created using proprietary data, such as Google software (Google Maps, Street View, and Earth). For more information, see our copyright guidelines: http://journals.plos.org/plosone/s/licenses-and-copyright.

We require you to either (1) present written permission from the copyright holder to publish these figures specifically under the CC BY 4.0 license, or (2) remove the figures from your submission:

1. You may seek permission from the original copyright holder of Figures 1,5 and 6 to publish the content specifically under the CC BY 4.0 license.

We recommend that you contact the original copyright holder with the Content Permission Form (http://journals.plos.org/plosone/s/file?id=7c09/content-permission-form.pdf) and the following text:

“I request permission for the open-access journal PLOS ONE to publish XXX under the Creative Commons Attribution License (CCAL) CC BY 4.0 (http://creativecommons.org/licenses/by/4.0/). Please be aware that this license allows unrestricted use and distribution, even commercially, by third parties. Please reply and provide explicit written permission to publish XXX under a CC BY license and complete the attached form.”

Please upload the completed Content Permission Form or other proof of granted permissions as an 'Other' file with your submission.

In the figure caption of the copyrighted figure, please include the following text: “Reprinted from [ref] under a CC BY license, with permission from [name of publisher], original copyright [original copyright year].”

2. If you are unable to obtain permission from the original copyright holder to publish these figures under the CC BY 4.0 license or if the copyright holder’s requirements are incompatible with the CC BY 4.0 license, please either i) remove the figure or ii) supply a replacement figure that complies with the CC BY 4.0 license. Please check copyright information on all replacement figures and update the figure caption with source information. If applicable, please specify in the figure caption text when a figure is similar but not identical to the original image and is therefore for illustrative purposes only.

The following resources for replacing copyrighted map figures may be helpful:

USGS National Map Viewer (public domain): http://viewer.nationalmap.gov/viewer/

The Gateway to Astronaut Photography of Earth (public domain): http://eol.jsc.nasa.gov/sseop/clickmap/

Maps at the CIA (public domain): https://www.cia.gov/library/publications/the-world-factbook/index.html and https://www.cia.gov/library/publications/cia-maps-publications/index.html

NASA Earth Observatory (public domain): http://earthobservatory.nasa.gov/

Landsat: http://landsat.visibleearth.nasa.gov/

USGS EROS (Earth Resources Observatory and Science (EROS) Center) (public domain): http://eros.usgs.gov/#

Natural Earth (public domain): http://www.naturalearthdata.com/

7. Please ensure that you refer to Figure 1 in your text as, if accepted, production will need this reference to link the reader to the figure.

8. Please include a caption for figure 7.

9. Please include a copy of Table 2 which you refer to in your text on page 5.

10. If the reviewer comments include a recommendation to cite specific previously published works, please review and evaluate these publications to determine whether they are relevant and should be cited. There is no requirement to cite these works unless the editor has indicated otherwise.

[Note: HTML markup is below. Please do not edit.]

Reviewers' comments:

Reviewer's Responses to Questions

**Comments to the Author**

1. Is the manuscript technically sound, and do the data support the conclusions?

Reviewer #1: Yes

Reviewer #2: Yes

**********

2. Has the statistical analysis been performed appropriately and rigorously?

Reviewer #1: No

Reviewer #2: Yes

**********

3. Have the authors made all data underlying the findings in their manuscript fully available?

The PLOS Data policy

Reviewer #1: Yes

Reviewer #2: Yes

**********

4. Is the manuscript presented in an intelligible fashion and written in standard English?

Reviewer #1: Yes

Reviewer #2: Yes

**********

Reviewer #1: Introduction

1. Why authors have chosen Biomod2 over other alternative modeling frameworks. Justify it.

2. Authors should abbreviate species names after the first mention. For example, it is acceptable to use Xanthium strumarium the first time, but in subsequent mentions, the abbreviated form X. strumarium should be used consistently throughout the manuscript

3. The dual role of X. strumarium is mentioned in the introduction; however, the specific challenges it poses in China—such as its impact on agriculture—are not sufficiently detailed. Additionally, while its medicinal value is briefly referenced, this aspect could be further elaborated to enhance the context and significance of the study.

Material and methods

1. Line 108, Provide rationale why resampling to 5 km was necessary.

2. Table 1, why specific environmental variables such as slope and NDVI were chosen for the analysis. Clarify, how these are relavant to Xanthium strumarium.

3. Ensure that all figures and tables are cited consistently and placed logically within the text. Notably, Figure 1 is not mentioned or referenced in the manuscript

4. Line 143, Briefly justify the selection of the 12 algorithms and list all 12 algorithms by their full name and acronym.

5. Clarify the absence of Table 2 in the manuscript and address the duplication of Table 3. Ensure that all tables are correctly numbered, referenced in the text, and presented in a logical sequence.

6. Authors should consider about adding a methodological flow diagram for better understanding.

Results

1. Line 189, Define the full names of acronyms when they first appear to ensure clarity. (e.g., FDA, MARS)

2. Line 213, The choice of the EMmean method is mentioned in the manuscript but not explained. Justify why this method is preferred over other methods.

3. Figure 3 includes AUC, Kappa, and TSS values. While AUC and Kappa are discussed in detail, TSS is not addressed in the results interpretation. Please either include a comprehensive discussion of the TSS results or clarify the reason for its omission to maintain consistency and completeness in the evaluation metrics.

4. Some figures are not self-explanatory. Captions are brief and lack units or explanations. (e.g., Make sure that colour gradient in figure 5 is clearly explained in the caption)

5. Provide a brief description about figure 5 and 6 in the text.

6. Legend of Figure 7 is missing.

7. The results lack statistical tests to validate differences between microbial communities or habitat prediction in section 3.5. Use ANOVA/PERMANOVA or any other method to statistically compare microbial diversity and habitat suitability across groups and scenarios.

Discussion

1. Discuss about the role of extreme weather events (e.g., floods) in shaping the distribution and competitive dynamics of X. strumarium.

2. Human activity is identified as the dominant environmental variable influencing X. strumarium, but its role is not adequately explained.

3. “Role of Rhizosphere Microbial Communities in Adaptation” section of the discussion could be improved by addressing how microbial interactions may shift in response to climate change.

4. While the role of microbial communities in adaptation is highlighted, the mechanisms by which specific microbial phyla contribute to plant adaptation remain underexplored. Elaborate on the functional roles of key microbial groups like Verrucomicrobia and Nitrospira.

5. Figures and quantitative data are not sufficiently integrated into the discussion.

General Comments

1. Split Results and Discussion clearly. Some interpretation slips into Results.

2. Several sentences are grammatically incorrect; a thorough proofreading is recommended to improve the clarity.

3. Figures are not self-explanatory. Legends are brief, kindly expands it. Also increase the resolution of the figures.

4. Species names must be italicized throughout the manuscript.

Reviewer #2: 1. Which climatic variables (temperature, precipitation, seasonality) most significantly influence the current and future distribution of X. strumarium?

2. Does climate-induced range expansion of X. strumarium correlate with shifts in its associated microbiome diversity and function?

**********

what does this mean?). If published, this will include your full peer review and any attached files.

If you choose “no”, your identity will remain anonymous but your review may still be made public.

**Do you want your identity to be public for this peer review?**  For information about this choice, including consent withdrawal, please see our Privacy Policy

Reviewer #1: No

Reviewer #2: No

**********

[NOTE: If reviewer comments were submitted as an attachment file, they will be attached to this email and accessible via the submission site. Please log into your account, locate the manuscript record, and check for the action link "View Attachments". If this link does not appear, there are no attachment files.]

While revising your submission, please upload your figure files to the Preflight Analysis and Conversion Engine (PACE) digital diagnostic tool, https://pacev2.apexcovantage.com/. PACE helps ensure that figures meet PLOS requirements. To use PACE, you must first register as a user. Registration is free. Then, login and navigate to the UPLOAD tab, where you will find detailed instructions on how to use the tool. If you encounter any issues or have any questions when using PACE, please email PLOS at figures@plos.org. Please note that Supporting Information files do not need this step.

Attachment Submitted filename: Comments on Xanthium strumarium.docx

# Author comment: Author response to Decision Letter 1

**DOI:** 10.1371/journal.pone.0351471.r002

26 Sep 2025

Response to Reviewers

Manuscript ID: PONE-D-25-30270

Title: Climate Change Impacts on Xanthium strumarium Distribution: Integrating Species Distribution Models with Rhizosphere Microbiome Analysis in China

Dear Academic Editor and Reviewers,

Thank you for your valuable feedback and thoughtful comments on our manuscript, which have helped us to significantly improve its quality. We have carefully addressed each point raised and have revised the manuscript accordingly. Our point-by-point responses are detailed below. All changes made in the manuscript are highlighted with track changes in the revised file.

Part A: Responses to Journal Requirements

We have carefully reviewed and revised the manuscript to comply with PLOS ONE's style requirements, including those for formatting and file naming. We have added a statement in the “Materials and Methods” section (Section 2.3) to clarify that no specific permits were required for the field sampling. The soil samples were collected from public-access areas in Inner Mongolia, China, and did not involve any protected species or private lands.

The revised statement is: “The Inner Mongolia Autonomous Region Natural Science Foundation Project (2023LHMS03007) provided funding for the article processing charges (APC) of this publication. The funder had no role in the study design, data collection and analysis, decision to publish, or preparation of the manuscript.”

We confirm that all relevant data necessary to replicate the study's findings are included within the manuscript and its Supporting Information files.

The corresponding author's ORCID iD has been created and validated in the Editorial Manager system.

We apologize for the oversight regarding the copyrighted maps. We confirm that Figures 1, 6, and 7 (the originally referenced Fig 5 and 6) were produced using standard map data from the official standard map service of the Ministry of Natural Resources of China, which are publicly available for scientific use. We have added a specific statement to the captions of these figures to reflect this, including the official map approval number GS(2023)2767. This demonstrates that the base maps are officially approved and publicly accessible. The revised captions for these figures are as follows:

Fig 1: An integrated methodological framework for analyzing climate change impacts on Xanthium strumarium distribution and its rhizosphere microbiome. The geographical components within this figure are based on standard map data from the official standard map service of the Ministry of Natural Resources of China (map approval number GS(2023)2767), used without modification.

Fig 6: Predicted potential distribution of suitable habitats for Xanthium strumarium in China under current environmental conditions. The base map for this figure was generated using standard map data from the official standard map service of the Ministry of Natural Resources of China (map approval number GS(2023)2767), with the geographical elements used without modification.

Fig 7: Projected future distributions of suitable habitats for Xanthium strumarium in China. The base map for this figure was generated using standard map data from the official standard map service of the Ministry of Natural Resources of China (map approval number GS(2023)2767), with the geographical elements used without modification.

We have revised the manuscript to ensure that Figure 1 is correctly cited and placed logically within the text in the “Materials and Methods” section. We have also added a detailed caption for Figure 7, which was previously missing. We apologize for the confusion and have now added the missing Table 2 (Rhizosphere soil sampling sites) to the manuscript, and all subsequent tables have been renumbered to ensure a logical sequence. No specific citations were recommended by the reviewer, so no action was required here.

Part B: Responses to Reviewer Comments

Reviewer #1

Introduction

Justify Biomod2 selection. We have revised the Introduction to provide a clear justification for choosing the Biomod2 framework. We highlight its ability to integrate multiple modeling algorithms, which reduces the uncertainty inherent in single models and provides a more robust and comprehensive prediction.

Abbreviate species names. We have thoroughly reviewed the manuscript and have consistently abbreviated Xanthium strumarium to X. strumarium after its first mention in the abstract and introduction.

Elaborate on the dual role of X. strumarium. We have expanded the Introduction to provide more detailed context on the dual nature of X. strumarium. We have added specific examples of its negative impact on Chinese agriculture, such as its invasion of farmlands and contamination of crops and livestock products, to enhance the significance of the study.

Materials and Methods

Rationale for 5 km resampling. We have added a sentence in the “Data Sources and Processing” section to explain the rationale for selecting a 5 km resolution. This scale was chosen to ensure methodological consistency, ecological appropriateness for a widespread species, and to minimize noise and spatial inaccuracies in the occurrence data.

Justify selection of variables. We have clarified the relevance of specific environmental variables, such as slope and NDVI, to X. strumarium in the “Data Sources and Processing” section. We explain that these variables are ecologically relevant to a ruderal species that thrives in disturbed and low-gradient environments.

Consistent figure and table citations. We have corrected all figure and table citations to ensure they are consistent and logically placed within the text.

Justify and list 12 algorithms. We have corrected the number of algorithms from 12 to 11, as Support Vector Machine (SVM) was initially part of the framework but was excluded. We apologize for this error. We have updated the text in “Models Construction and Changes in Ecological Niches” to clearly state that 11 algorithms were used. The full names and acronyms of all 11 algorithms are already listed in the manuscript. We have also added a brief justification for using an ensemble of diverse algorithms, explaining that this approach captures a wide range of species-environment relationships and reduces uncertainty.

Clarify table numbering. We apologize for the confusion. We have added the missing Table 2 (Rhizosphere soil sampling sites) to the manuscript, and all subsequent tables have been renumbered to ensure a logical sequence.

Add a methodological flow diagram. We have added a methodological flow diagram (Fig 1) to the “Materials and Methods” section, which visually summarizes the entire research process.

Results

Define acronyms. The full names of all acronyms, including FDA and MARS, are defined in the text when they first appear. We have reviewed the manuscript to ensure this consistency.

Justify the EMmean method. As requested in the provided document, we have already added a comprehensive justification for the choice of the EMmean method, highlighting its robustness, transparency, and ability to balance predictions from diverse algorithms.

Discuss TSS values. We have added a sentence to the “Model Precision Assessment” section to briefly interpret the TSS values, mentioning that the RF model achieved the highest score and that several other models also performed well, providing a consistent analysis of all three metrics (AUC, TSS, and Kappa).

Improve figure captions. We have expanded the captions for Figures 4, 5, 6, 7, 8, 9, 10, 11, 12, and 13 to be more self-explanatory. We have also added units and clearer explanations for color gradients and other visual elements, ensuring readers can understand the figures without referring to the main text.

Provide a brief description about figures 5 and 6 in the text. We have revised the “Bioclimatic Variable Contribution” and “Present Potential Geographic Spread” sections to include brief descriptions and references to Figures 5 and 6, respectively.

Legend for Figure 7. We have added a clear and descriptive legend for Figure 7.

Add statistical tests for microbial communities. We apologize for this oversight. We have now performed a PERMANOVA (Adonis) analysis to statistically compare the microbial community composition among the different sampling sites. We have added the results of this analysis to the “Rhizosphere Microbial Abundance” section (Section 3.5), including the p-values and R² values, which now statistically validate the observed differences.

Discussion

Role of extreme weather events. We have added a sentence to the “Impact of Climate Change on Habitat Distribution” section to discuss the role of extreme weather events, such as floods, in creating disturbed habitats that promote the spread and competitive advantage of X. strumarium.

Clarify the role of human activity. We have expanded the discussion on the dominant role of the human footprint. We now explicitly state that the human footprint index captures the cumulative impact of land-use changes like agricultural expansion and urbanization, which create the disturbed environments where this species thrives. We have also integrated the quantitative data from Fig 5 to highlight its significant contribution (66.6%) to the model.

Microbial interactions and climate change. We have added a sentence to the “Role of Rhizosphere Microbial Communities in Adaptation” section to discuss how microbial interactions may shift in response to climate change, specifically mentioning that altered soil moisture and temperature could lead to an increase in stress-tolerant microbial taxa.

Functional roles of Verrucomicrobia and Nitrospira. We have added a detailed explanation of the functional roles of Verrucomicrobia and Nitrospira, clarifying their contributions to nitrogen cycling and the degradation of complex organic matter. We also linked these functions to the overall 'Metabolism' pathway predicted by Tax4Fun to provide a deeper mechanistic insight.

Integrate figures and data. We have thoroughly reviewed the Discussion section and have integrated quantitative data and direct references to Figures 5, 8, 9, and 11 to support our interpretations and conclusions, ensuring the discussion is well-supported by the results.

General Comments

Split Results and Discussion. We have re-evaluated the manuscript and have split the “Results” and “Discussion” sections more clearly. We have moved all interpretive sentences to the Discussion and ensured that the Results section focuses solely on presenting the data and findings.

Proofreading. The manuscript has undergone a thorough proofreading to correct grammatical errors and improve the overall clarity and readability.

Figure clarity and resolution. As stated in our response to Reviewer 1's comments on the Results section, we have expanded all figure captions to make them more self-explanatory. We have also ensured that all figures meet the specified resolution requirements for publication.

Italicize species names. We have performed a final check to ensure that all species names (Xanthium strumarium and X. strumarium) are consistently italicized throughout the manuscript.

We believe that these revisions have significantly improved the quality and rigor of our manuscript. We are confident that it now meets the publication criteria for PLOS ONE. We look forward to your positive decision.

Sincerely,

Jinyang Dong & Liyan Zhang

Co-first authors

Attachment Submitted filename: Response to Reviewers.docx

# Peer review: Decision Letter 1

**DOI:** 10.1371/journal.pone.0351471.r003

**Authors:** Debasis Mitra

30 Dec 2025

Dear Dr. Jiang,

Thank you for submitting your manuscript to PLOS ONE. After careful consideration, we feel that it has merit but does not fully meet PLOS ONE’s publication criteria as it currently stands. Therefore, we invite you to submit a revised version of the manuscript that addresses the points raised during the review process.

Please submit your revised manuscript by Feb 13 2026 11:59PM. If you will need more time than this to complete your revisions, please reply to this message or contact the journal office at plosone@plos.org. When you're ready to submit your revision, log on to https://www.editorialmanager.com/pone/ and select the 'Submissions Needing Revision' folder to locate your manuscript file.

- A letter that responds to each point raised by the academic editor and reviewer(s). You should upload this letter as a separate file labeled 'Response to Reviewers'.
- A marked-up copy of your manuscript that highlights changes made to the original version. You should upload this as a separate file labeled 'Revised Manuscript with Track Changes'.
- An unmarked version of your revised paper without tracked changes. You should upload this as a separate file labeled 'Manuscript'.

If you would like to make changes to your financial disclosure, please include your updated statement in your cover letter. Guidelines for resubmitting your figure files are available below the reviewer comments at the end of this letter.

If applicable, we recommend that you deposit your laboratory protocols in protocols.io to enhance the reproducibility of your results. Protocols.io assigns your protocol its own identifier (DOI) so that it can be cited independently in the future. For instructions see: https://journals.plos.org/plosone/s/submission-guidelines#loc-laboratory-protocols. Additionally, PLOS ONE offers an option for publishing peer-reviewed Lab Protocol articles, which describe protocols hosted on protocols.io. Read more information on sharing protocols at https://plos.org/protocols?utm_medium=editorial-email&utm_source=authorletters&utm_campaign=protocols.

We look forward to receiving your revised manuscript.

Kind regards,

Debasis Mitra

Academic Editor

PLOS One

Journal Requirements:

1. If the reviewer comments include a recommendation to cite specific previously published works, please review and evaluate these publications to determine whether they are relevant and should be cited. There is no requirement to cite these works unless the editor has indicated otherwise.

2. Please review your reference list to ensure that it is complete and correct. If you have cited papers that have been retracted, please include the rationale for doing so in the manuscript text, or remove these references and replace them with relevant current references. Any changes to the reference list should be mentioned in the rebuttal letter that accompanies your revised manuscript. If you need to cite a retracted article, indicate the article’s retracted status in the References list and also include a citation and full reference for the retraction notice.

[Note: HTML markup is below. Please do not edit.]

Reviewers' comments:

Reviewer's Responses to Questions

**Comments to the Author**

Reviewer #3: (No Response)

**********

2. Is the manuscript technically sound, and do the data support the conclusions?

Reviewer #3: Yes

**********

3. Has the statistical analysis been performed appropriately and rigorously?

Reviewer #3: (No Response)

**********

4. Have the authors made all data underlying the findings in their manuscript fully available?

The PLOS Data policy

Reviewer #3: (No Response)

**********

5. Is the manuscript presented in an intelligible fashion and written in standard English?

Reviewer #3: Yes

**********

Reviewer #3: (No Response)

**********

what does this mean?). If published, this will include your full peer review and any attached files.

If you choose “no”, your identity will remain anonymous but your review may still be made public.

**Do you want your identity to be public for this peer review?**  For information about this choice, including consent withdrawal, please see our Privacy Policy

Reviewer #3: No

**********

[NOTE: If reviewer comments were submitted as an attachment file, they will be attached to this email and accessible via the submission site. Please log into your account, locate the manuscript record, and check for the action link "View Attachments". If this link does not appear, there are no attachment files.]

To ensure your figures meet our technical requirements, please review our figure guidelines: https://journals.plos.org/plosone/s/figures

You may also use PLOS’s free figure tool, NAAS, to help you prepare publication quality figures: https://journals.plos.org/plosone/s/figures#loc-tools-for-figure-preparation.

NAAS will assess whether your figures meet our technical requirements by comparing each figure against our figure specifications.

Attachment Submitted filename: comment.docx

# Author comment: Author response to Decision Letter 2

**DOI:** 10.1371/journal.pone.0351471.r004

27 Jan 2026

Thank you very much for giving us the opportunity to revise our manuscript. We sincerely appreciate the time and effort you and the reviewers have dedicated to providing insightful feedback on our work.

We have carefully considered the comments raised by Reviewer #3 (as contained in the attachment). We found the suggestions extremely constructive and have addressed each point in detail. The manuscript has been revised to incorporate invasion risk zoning, discussions on soil chemical properties, and updated literature from 2023–2025.

Below is a point-by-point response to the specific comments.

Response to Reviewer #3

Comment 1: "The manuscript was written well but some modifications are required. Xanthium strumarium, an invasive species... In the abstract part, potential risk zones may be included for better understanding."

Response: We appreciate this suggestion. We have revised the Abstract to explicitly identify high-risk areas. The text now reads: "Future climate scenarios predict habitat expansion of 8.9–28.6%, identifying high-risk invasion zones primarily concentrated in Yunnan, Guangdong, and Inner Mongolia provinces." This modification provides a clearer warning for management purposes.

Comment 2: "It was observed that to investigate the potential impacts of climate change on the distribution of X. strumarium, species distribution model was used. It may be more better if the model predict No Risk Zones, Low-Risk Zones, Medium Risk Zones, High-Risk Zones etc."

Response: We fully agree that classifying results into risk zones is more practical for invasion management.

Methodology Update: In Section 2.2, we reclassified the habitat suitability indices into four discrete categories: No-Risk Zone (0–0.25), Low-Risk Zone (0.25–0.50), Medium-Risk Zone (0.50–0.75), and High-Risk Zone (0.75–1.0).

Visuals Updated: We have updated Figure 6, Figure 7, and Table 3 to reflect this new terminology.

Comment 3: "During the analysis of distribution pattern, soil chemical properties like CEC, soil available N etc maybe include."

Response: This is a critical point. While our broad-scale SDM relied on climatic and topographic variables due to the limitations of high-resolution soil raster data at the national scale, we have significantly strengthened the discussion on soil chemical properties by linking them to our Human Footprint and Microbiome results.

In Section 4.1: We expanded the discussion to explain that the high contribution of the "Human Footprint" variable serves as a proxy for nutrient-rich soil conditions caused by anthropogenic disturbance. We explicitly discussed how CEC and Soil Available Nitrogen act as drivers for this nitrophilous species, citing recent evidence.

In Section 4.2: We utilized our molecular data to provide biological validation that soil nitrogen cycling is indeed a key limiting factor for X. strumarium, effectively bridging the gap between macro-models and micro-soil properties.

Comment 4: "Discussion part maybe enriched with more recent evidences as well as recent references."

Response: We have extensively updated the Discussion section with literature published between 2023 and 2025 to ensure the manuscript reflects the state-of-the-art.

Mechanisms of Invasion: We incorporated recent studies on how disturbance releases nutrients.

Microbiome Adaptation: We added references regarding how invasive plants actively reshape microbial communitie.

Soil Heterogeneity: We cited recent findings on soil nutrient heterogeneity.

Journal Requirements regarding References: We have reviewed our reference list to ensure completeness and correctness. We confirm that no retracted papers have been cited.

We believe that these revisions have significantly improved the quality and clarity of the manuscript.

Attachment Submitted filename: Response to Reviewers_20260127.docx

# Editor report: Decision Letter 2

**DOI:** 10.1371/journal.pone.0351471.r005

**Authors:** Fei Xu

12 Apr 2026

Dear Dr. Jiang,

Thank you for submitting your manuscript to PLOS ONE. After careful consideration, we feel that it has merit but does not fully meet PLOS ONE’s publication criteria as it currently stands. Therefore, we invite you to submit a revised version of the manuscript that addresses the points raised during the review process.

Please submit your revised manuscript by May 27 2026 11:59PM. If you will need more time than this to complete your revisions, please reply to this message or contact the journal office at plosone@plos.org. When you're ready to submit your revision, log on to https://www.editorialmanager.com/pone/ and select the 'Submissions Needing Revision' folder to locate your manuscript file.

- A letter that responds to each point raised by the academic editor and reviewer(s). You should upload this letter as a separate file labeled 'Response to Reviewers'.
- A marked-up copy of your manuscript that highlights changes made to the original version. You should upload this as a separate file labeled 'Revised Manuscript with Track Changes'.
- An unmarked version of your revised paper without tracked changes. You should upload this as a separate file labeled 'Manuscript'.

If you would like to make changes to your financial disclosure, please include your updated statement in your cover letter. Guidelines for resubmitting your figure files are available below the reviewer comments at the end of this letter.

If applicable, we recommend that you deposit your laboratory protocols in protocols.io to enhance the reproducibility of your results. Protocols.io assigns your protocol its own identifier (DOI) so that it can be cited independently in the future. For instructions see: https://journals.plos.org/plosone/s/submission-guidelines#loc-laboratory-protocols. Additionally, PLOS ONE offers an option for publishing peer-reviewed Lab Protocol articles, which describe protocols hosted on protocols.io. Read more information on sharing protocols at https://plos.org/protocols?utm_medium=editorial-email&utm_source=authorletters&utm_campaign=protocols.

As the corresponding author, your ORCID iD is verified in the submission system and will appear in the published article. PLOS supports the use of ORCID, and we encourage all coauthors to register for an ORCID iD and use it as well. Please encourage your coauthors to verify their ORCID iD within the submission system before final acceptance, as unverified ORCID iDs will not appear in the published article. *Only*  the individual author can complete the verification step; PLOS staff *cannot*  verify ORCID iDs on behalf of authors.

We look forward to receiving your revised manuscript.

Kind regards,

Fei Xu, Ph.D.

Academic Editor

PLOS One

Journal Requirements:

If the reviewer comments include a recommendation to cite specific previously published works, please review and evaluate these publications to determine whether they are relevant and should be cited. There is no requirement to cite these works unless the editor has indicated otherwise.

Additional Editor Comments:

The authors are requested to clarify several questionable points:

1.The reasons for the decrease, instead of increase, in the areas of high-risk zones as shown in Table 7;

2.The issue of potential mirrored image in the results of niche stability under the SSP5-8.5 scenario in fig. 8;

3.The selection of microbial sampling sites—specifically, why these five locations were chosen? The lack of connection between the sites and the predictive model, and the absence of integration with environmental factors in the analysis process, make it difficult to support the conclusions about the changes in species distribution .

[Note: HTML markup is below. Please do not edit.]

[NOTE: If reviewer comments were submitted as an attachment file, they will be attached to this email and accessible via the submission site. Please log into your account, locate the manuscript record, and check for the action link "View Attachments". If this link does not appear, there are no attachment files.]

To ensure your figures meet our technical requirements, please review our figure guidelines: https://journals.plos.org/plosone/s/figures

You may also use PLOS’s free figure tool, NAAS, to help you prepare publication quality figures: https://journals.plos.org/plosone/s/figures#loc-tools-for-figure-preparation.

NAAS will assess whether your figures meet our technical requirements by comparing each figure against our figure specifications.

# Author comment: Author response to Decision Letter 3

**DOI:** 10.1371/journal.pone.0351471.r006

24 May 2026

Dear Reviewers,

Thank you for giving us the opportunity to submit a revised draft of our manuscript. We deeply appreciate the time and effort that you and the reviewers have dedicated to providing valuable, constructive, and insightful feedback. We have carefully read all the comments and found them immensely helpful in improving the quality, logical rigor, and clarity of our study.

In this revised manuscript, we have addressed all the concerns raised by the reviewers. Specifically, the most significant improvements include:

Clarifying the Spatial Logic: We have explicitly detailed the rationale for selecting the five microbial sampling sites in Inner Mongolia, seamlessly bridging our macro-scale Species Distribution Model (SDM) predictions (specifically the Human Footprint Index) with our micro-scale microbiome findings.

Explaining Ecological Trends: We have provided a robust ecological interpretation for the projected decrease in the high-risk zones of Xanthium strumarium under future high-emission scenarios (the "suitability decay" phenomenon).

Refining Conclusions: We have carefully calibrated our language to acknowledge the spatial limitations of the microbial sampling, presenting the findings as vital mechanistic insights rather than definitive nationwide proofs.

Following this summary, a detailed, point-by-point response to each of the comments raised by the editor and reviewers is presented below.

Comment 1: The reasons for the decrease, instead of increase, in the areas of high-risk zones as shown in Table 7.

Response: We deeply appreciate the reviewer's rigorous review and insightful observation. First, we would like to gently clarify that the projected changes in the distribution areas, including the high-risk zones, are detailed in Table 3 and Figure 7 of our manuscript (we respectfully assume the mention of Table 7 was a minor typographical oversight).

The decrease in high-risk zones under future scenarios—particularly under the high-emission SSP5-8.5 scenario—is indeed a counterintuitive but ecologically plausible phenomenon, rather than a model artifact. This non-linear trend, which we refer to as "suitability decay," can be attributed to the following key factors:

Physiological Thresholds: Under extreme warming scenarios, temperature and moisture stress in the current core habitats may exceed the optimal physiological limits for X. strumarium. As environmental conditions drift beyond these optimal thresholds, the habitat quality in these historic core areas degrades from "High-Risk" down to "Medium- or Low-Risk."

Niche Fragmentation and Range Shift: While the overall total suitable area expands (specifically, driven by a significant increase in Low- and Medium-Risk zones as shown in Table 3), the highly concentrated optimal "hotspots" undergo spatial fragmentation. The species is simultaneously colonizing new, marginal environments at higher latitudes or altitudes, which initially present as lower-risk zones.

Environmental Trade-offs: Our model indicates that the Human Footprint Index is the dominant driver (contributing 66.6%). While anthropogenic disturbance provides the necessary fertile micro-environments, the intensifying macro-climatic stressors in future scenarios counterbalance these benefits. This trade-off reduces the overall probability of maintaining "optimal" (high-risk) suitability in the original infestation centers.

Action taken: To ensure this phenomenon is clearly understood by readers, we have explicitly expanded upon this "suitability decay" and its implications for broader invasion management in the revised Discussion (Section 4.1).

Comment 2: Potential mirroring issues in the niche stability results under the SSP5-8.5 scenario (as shown in Figure 8).

Response: We respectfully disagree with the reviewer’s interpretation regarding a "mirroring issue." We would like to clarify that the asymmetrical protrusion observed in the bottom-left corner of the PCA space under the SSP5-8.5 scenarios (both 2041–2060 and 2061–2080) is not a graphical glitch, code error, or mirroring artifact. In the niche analysis performed via the ecospat package, the solid contours (red and green lines) delineate the outer boundaries of the available background climate space (environmental envelope) for the respective periods. The specific shape and extension of these lines are driven entirely by the underlying climate data:

Real Climate Shifts under Extreme Scenarios: The SSP5-8.5 is an extreme, high-emission scenario characterized by severe climate anomalies. The distinct "tail" or loop protruding into the negative PC1 and PC2 space explicitly reflects a dramatic shift and expansion of the macro-climatic baseline (e.g., extreme temperature/precipitation combinations or the emergence of non-analog novel climates) that occurs only under severe warming.

Evidence from Mild Scenarios: As shown in Figure 8, this protrusion is completely absent in the milder scenarios (SSP1-2.6 and SSP2-4.5), where the background climate space remains stable and symmetrical. If this were a computational or mirroring bug within the plotting script, it would consistently manifest across all climate scenarios. Its exclusive appearance under SSP5-8.5 confirms that the model is accurately visualizing the dramatic distortion of the future environmental space.

Action taken: To prevent any future misinterpretation by readers, we have added a clarifying statement in the Figure 8 caption and the corresponding Results section to explicitly state that these extended contours represent the expansion of extreme background climate spaces under the high-emission scenario.

Comment 3: The selection of microbial sampling sites—specifically, why these five locations were chosen? The lack of connection between the sites and the predictive model, and the absence of integration with environmental factors in the analysis process, make it difficult to support the conclusions about the changes in species distribution.

Response: We sincerely appreciate the reviewer’s critical and insightful comment. We agree that bridging the gap between macro-scale predictive models (SDM) and micro-scale ecological data (microbiome) requires a highly explicit logical connection. We have thoroughly revised our manuscript to clarify the strategic rationale behind our site selection and to better integrate environmental factors into our interpretation.

We have addressed this concern from the following three dimensions:

1. Strategic Site Selection Driven by SDM Predictions: The choice of Inner Mongolia and these five specific habitats was not arbitrary, but rather a targeted validation of our preliminary SDM results. First, our Biomod2 model identified Inner Mongolia as a highly vulnerable, emerging hotspot for Xanthium strumarium invasion under future climate change scenarios. Sampling at the invasion front allows us to capture the early adaptive mechanisms of expanding populations.

2. Establishing the Link: Anthropogenic Disturbance Gradient: The predictive model revealed that the Human Footprint Index (HFI) is the single most dominant macro-environmental driver of X. strumarium distribution, contributing 66.6% to the model. To directly connect our field sampling with this key environmental factor, we deliberately selected five habitats that represent a clear gradient of anthropogenic disturbance: Urban Green Space and Farmland Edge (High Disturbance) vs. Grassland, Montane Forest, and Temperate Steppe (Low to Moderate Disturbance). This allows us to investigate how macro-environmental filtering (human activities) translates into micro-ecological responses.

3. Integration of Environmental Factors and Mechanistic Insights: In the revised Discussion, we have deeply integrated these macro-environmental factors with the microbial functions. Anthropogenic disturbances typically lead to localized soil nutrient shifts, particularly nitrogen accumulation. Our microbiome data provides a direct mechanistic explanation for the SDM: in high-disturbance sites (high HFI), X. strumarium exhibits a distinct rhizosphere assembly, significantly enriching nitrogen-cycling and stress-tolerant taxa (such as Nitrospira and Pseudomonas). This micro-level microbial recruitment provides the physiological support needed for the plant to thrive in the highly suitable habitats predicted by the macro-model.

4. Calibrating the Scope of Our Conclusions: We acknowledge the spatial scale limitation raised by the reviewer—five localized sampling sites cannot fully represent nationwide species distribution shifts. Therefore, we have carefully toned down our conclusions. In the revised text, we clarify that the microbiome data serves as a localized mechanistic insight into how the species adapts to disturbance regimes, rather than a direct nationwide proof. We have also explicitly added this spatial scale mismatch as an inherent limitation in the "Uncertainties" section (Section 4.3).

Attachment Submitted filename: letter.docx

# Editor report: Decision Letter 3

**DOI:** 10.1371/journal.pone.0351471.r007

**Authors:** Fei Xu

27 May 2026

Climate Change Impacts on Xanthium strumarium Distribution: Integrating Species Distribution Models with Rhizosphere Microbiome Analysis in China

PONE-D-25-30270R3

Dear Dr. Jiang,

We’re pleased to inform you that your manuscript has been judged scientifically suitable for publication and will be formally accepted for publication once it meets all outstanding technical requirements.

Within one week, you’ll receive an e-mail detailing the required amendments. When these have been addressed, you’ll receive a formal acceptance letter and your manuscript will be scheduled for publication.

An invoice will be generated when your article is formally accepted. Please note, if your institution has a publishing partnership with PLOS and your article meets the relevant criteria, all or part of your publication costs will be covered. Please make sure your user information is up-to-date by logging into Editorial Manager at Editorial Manager® and clicking the ‘Update My Information' link at the top of the page. For questions related to billing, please contact billing support.

If your institution or institutions have a press office, please notify them about your upcoming paper to help maximize its impact. If they’ll be preparing press materials, please inform our press team as soon as possible -- no later than 48 hours after receiving the formal acceptance. Your manuscript will remain under strict press embargo until 2 pm Eastern Time on the date of publication. For more information, please contact onepress@plos.org.

Kind regards,

Fei Xu, Ph.D.

Academic Editor

PLOS One

# Editor report: Acceptance letter

**DOI:** 10.1371/journal.pone.0351471.r008

**Authors:** Fei Xu

PONE-D-25-30270R3

PLOS One

Dear Dr. Jiang,

I'm pleased to inform you that your manuscript has been deemed suitable for publication in PLOS One. Congratulations! Your manuscript is now being handed over to our production team.

At this stage, our production department will prepare your paper for publication. This includes ensuring the following:

* All references, tables, and figures are properly cited

* All relevant supporting information is included in the manuscript submission,

* There are no issues that prevent the paper from being properly typeset

You will receive further instructions from the production team, including instructions on how to review your proof when it is ready. Please keep in mind that we are working through a large volume of accepted articles, so please give us a few days to review your paper and let you know the next and final steps.

Lastly, if your institution or institutions have a press office, please let them know about your upcoming paper now to help maximize its impact. If they'll be preparing press materials, please inform our press team within the next 48 hours. Your manuscript will remain under strict press embargo until 2 pm Eastern Time on the date of publication. For more information, please contact onepress@plos.org.

You will receive an invoice from PLOS for your publication fee after your manuscript has reached the completed accept phase. If you receive an email requesting payment before acceptance or for any other service, this may be a phishing scheme. Learn how to identify phishing emails and protect your accounts at https://explore.plos.org/phishing.

If we can help with anything else, please email us at customercare@plos.org.

Thank you for submitting your work to PLOS One and supporting open access.

Kind regards,

PLOS ONE Editorial Office Staff

on behalf of

Dr. Fei Xu

Academic Editor

PLOS One
